# Spatial and Species Variations of Bacterial Community Structure and Putative Function in Seagrass Rhizosphere Sediment

**DOI:** 10.3390/life11080852

**Published:** 2021-08-20

**Authors:** Juan Ling, Weiguo Zhou, Qingsong Yang, Jianping Yin, Jian Zhang, Qiuying Peng, Xiaofang Huang, Yuhang Zhang, Junde Dong

**Affiliations:** 1 CAS Key Laboratory of Tropical Marine Bio-Resources and Ecology, Guangdong Provincial Key Laboratory of Applied Marine Biology, South China Sea Institute of Oceanology, Chinese Academy of Sciences, Guangzhou 510301, China; lingjuan@scsio.ac.cn (J.L.); zhouweiguo@scsio.ac.cn (W.Z.); qsyang@scsio.ac.cn (Q.Y.); yjp@scsio.ac.cn (J.Y.); zhangjian196@mails.ucas.ac.cn (J.Z.); pengqiuying18@mails.ucas.edu.cn (Q.P.); huangxiaofang602@126.com (X.H.); zhangyuhang20@mails.ucas.ac.cn (Y.Z.); 2 Southern Marine Science and Engineering Guangdong Laboratory (Guangzhou), Guangzhou 511458, China; 3 Key Laboratory of Tropical Marine Biotechnology of Hainan Province, Sanya Institute of Oceanology, SCSIO, Sanya National Marine Ecosystem Research Station, Chinese Academy of Sciences, Sanya 572000, China; 4 Innovation Academy of South China Sea Ecology and Environmental Engineering, Chinese Academy of Sciences, Guangzhou 511458, China; 5 College of Earth and Planetary Sciences, University of Chinese Academy of Sciences, Beijing 100049, China

**Keywords:** seagrass, rhizosphere bacterial communities, community structure, functional groups, core microbial community, coral reef ecosystems

## Abstract

Seagrasses are an important part of the coral reef ecosystem, and their rhizosphere microbes are of great ecological importance. However, variations in diversity, composition, and potential functions of bacterial communities in the seagrass rhizosphere of coral reef ecosystems remain unclear. This study employed the high-throughput sequencing based on 16S rDNA gene sequences and functional annotation of prokaryotic taxa (FAPROTAX) analysis to investigate these variations based on seagrass species and sampling locations, respectively. Results demonstrated that the seagrass rhizosphere microbial community was mainly dominated by phylum Proteobacteria (33.47%), Bacteroidetes (23.33%), and Planctomycetes (12.47%), while functional groups were mainly composed of sulfate respiration (14.09%), respiration of sulfur compounds (14.24%), aerobic chemoheterotrophy (20.87%), and chemoheterotrophy (26.85%). Significant differences were evident in alpha diversity, taxonomical composition and putative functional groups based on seagrass species and sampling locations. Moreover, the core microbial community of all investigated samples was identified, accounting for 63.22% of all obtained sequences. Network analysis indicated that most microbes had a positive correlation (82.41%), and two module hubs (phylum Proteobacteria) were investigated. Furthermore, a significant positive correlation was found between the OTUs numbers obtained and the functional groups assigned for seagrass rhizosphere microbial communities (*p* < 0.01). Our result would facilitate future investigation of the function of seagrass rhizosphere microbes.

## 1. Introduction

Coral reef plays important roles in the marine ecosystem for their high biodiversity, productivity, and economic and ecological services to millions of people [1]. Their biogenic carbonate structures are accumulated over many years by calcifying organisms, including corals and algae [2]. However, climate change (e.g., ocean acidification and global warming) and human activities both exert direct and indirect effects on the coral reef locally and globally, leading to a wide decline in the coral reef ecosystem [3,4]. Seagrass meadows could provide habitats for corals to live within and recruit by providing food, shelter, and a nursing place [1]. Moreover, seagrass can also increase the seawater pH and the scleractinian coral calcification rate through high photosynthesis, purify the water, stabilize the sediment, and mitigate the climate by being the vital component of the blue carbon [5,6,7]. It may also reduce two-fold disease levels for adjacent reef-building corals by comparing with the corals at paired sites without adjacent seagrass meadows [8]. 

Seagrass holobiont could execute many functions that may benefit their hosts [9,10,11]. Its structure and function are key to seagrass productivity, health, and the biogeochemical cycle of carbon, nitrogen, and sulfur cycle [11,12,13]. The seagrass rhizosphere microbe refers to the microbe that inhabits in the narrow zone surrounding and could be influenced by seagrass roots [13,14]. Moreover, the rhizosphere microbiome plays crucial roles in affecting plant health, such as nutrient uptake, preventing colonization by pathogens, and modulating host immunity, etc. [11,13,15]. The predominant bacteria in the rhizosphere of seagrass *Zostera marina*, *Z.noltii*, and *Cymodocea nodosa* were Proteobacteria, Bacteroidetes, Chloroflexi, Planctomycetes, Actinobacteria, and Acidobacteria [16]. Moreover, their results also showed that rhizobiomes of different seagrass species from the same region exhibited no significant differences, while seagrass derived from distinct sampling locations had significant variations [16]. 

Sulfide is a highly toxic compound that can be taken up into seagrass roots due to the low oxygen level in the sediment, and is the one main reason that causes seagrass death globally [17,18]. The investigation by metagenomic analysis revealed that seagrass *Z. marina’s rhizosphere* harbored free-living forms of sulfur-oxidizing chemolithoautotrophic symbionts, which may contribute much to the survival of seagrass through detoxification of sulfide in seagrass rhizosphere [14]. Another essential functional microbial group was the nitrogen cycling microbe. For example, 27% of the total nitrogen demand for seagrass growth could be fulfilled by efficiently recycling organic nitrogen, nitrogen fixation, or other ways of the external source of nitrogen [19]. Biological nitrogen fixation in the phyllosphere of *Posidonia oceanica* of the Mediterranean Sea supplied the total N demand for seagrass *P. oceanica* growth [19]. Moreover, the associated microbes of the tropical seagrass *Halophila stipulacea* has also been reported to fix nitrogen in different conditions and has provided their required host nitrogen [20]. There existed a beneficial mutualistic relationship between the seagrasses plant and heterotrophic nitrogen-fixing microbes in the seagrass rhizosphere [21].

Core microbial community may play an essential role in the microbial community’s function from specified habits, and identifying the core microbiome is crucial for understanding the stable and consistent microbial species across different sampling locations [16,22]. The previous investigation showed that core microbes among rhizobiomes of varying seagrass species and the same seagrass species, collected from other sites and sulfur-related metabolism microbes, were a significant component of the core rhizobiome of seagrasses [16]. 

However, there was rare information about the taxonomic and phylogenetic composition in the coral reef ecosystems’ seagrass rhizosphere [23]. Therefore, we conducted this study to answer the following questions in the coral reef ecosystem: (1) are there significant spatial and species variations in the diversity and composition in the seagrass rhizosphere bacterial communities?; (2) what are the major functional groups of seagrass rhizosphere bacteria?; and (3) do seagrass rhizosphere microbial communities of different species, and discrete sampling locations share core communities? Hence, high-throughput sequencing of 16S rDNA and functional annotation of prokaryotic taxa (FAPROTAX) analysis was used in this study to investigate their community structure and potential functions.

## 2. Materials and Methods

### 2.1. Description of Sampling Location 

**Figure 1:**
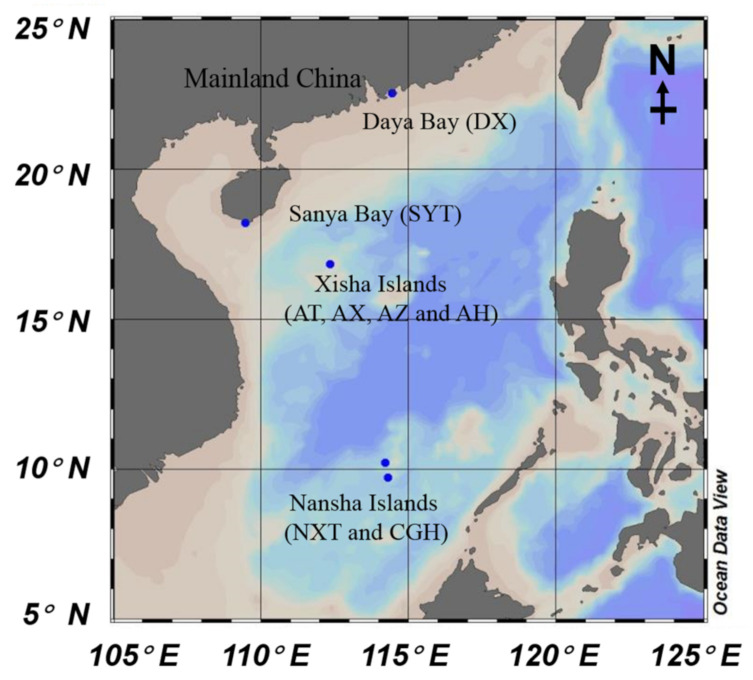
The sampling locations of the seagrass in four coral reef ecosystems with the samples in the parenthesis (DYH indicates seagrass *Halophila ovalis* from Daya Bay; SYT indicates seagrass *Thalassia* *hemprichii* from Sanya Bay, XST, XSH, XSC, and XSS indicate *T.* *hemprichii*, *H. ovalis*, *Cymodocea* *nodos* and *Syringodium* *isoetifolium* collected from the Xisha Islands, and NSH and NST indicates seagrass *H. ovalis* and *T.* *hemprichii* collected from the Nansha Islands). This map was created by Ocean Data View software 4.3.5 (http://odv.awi.de (accessed on 17 August 2020)).

Four coral reef ecosystems, i.e., Daya (DY) Bay, Luhuitou fringing reef of Sanya (SY) Bay, Xisha (XS) Islands, and Nansha (NS) Islands in the South China Sea across large spatial scales, were selected for seagrass rhizosphere microbe investigation (Figure 1). Seagrass forms dense meadows in all investigated coral reef ecosystems. Daya Bay, a shallow semi-closed bay (22.54° E, 114.45° N) with an area of ~600 km^2^, is under the typical subtropical marine climate. Numerous coral colonies distribute sporadically within the bay around the inner islands, such as Dalajia and Xiaolajia Islands. Seagrass *Halophila ovalis* (H) is the only species forming seagrass meadow in DY Bay. For the Luhuitou fringing reef of Sanya Bay (18.2° E, 109.47° N), seagrass *Thalassia* *hemprichii* (T) appeared following coral reef degradation, and it is the only seagrass species detected in this fringing reef. There are four seagrass species (*Cymodocea nodos* (C), *T. hemprichii*, *H. ovalis* (H), and *Syringodium isoetifolium* (S)) growing in one seagrass meadow in the Xisha Islands (XS) (16.84° E, 112.34° N). Besides, the Nansha Islands (NS) are in the southern South China Sea and under the effect of a typical tropical climate. Two seagrass species, *H. ovalis* and *T. hemprichii,* were collected from Nanxun Reef and Chigua Reef, respectively. The two species are the dominant seagrass species of where they are growing. The seagrass rhizosphere samples were named by combing the sampling locations and the seagrass species, e.g., DYH indicates the sample collected from Daya Bay and seagrass species is *H. ovalis*. Four investigated coral reef ecosystems are all under the effect of the East Asian monsoon (Northeast in the winter, and Southwest in the summer). The average atmospheric temperature in Sanya Bay was 30.74 °C, with warm summers (34.75 °C) and cold winters (27.20 °C) [24]. The temperature range and mean value for Daya Bay were 14.4–32.4 °C and the annual mean temperature was 22.4 °C, respectively [25]. The temperature range for Yongxing Island was from 15.5 to 37 °C, and the annual average temperature was 26.5 °C [26]. For the Nansha Islands, the temperature only slightly varies throughout the year, with an annual average of 28.1 °C [27].

### 2.2. Sample Collection and Physic-Chemical Property Measurement

Samples of seagrass rhizosphere sediment were collected in triplicate for each sample, and 24 samples in total were obtained. Sediment collection and physic-chemical property measurements were performed, according to Ling et al. [23]. Environmental parameters, e.g., pH, salinity, and DO, were simultaneously measured in situ. The overlying seawater column’s temperature and salinity above the seagrass meadows were measured by the YSI 6600V2 water quality sonde^TM^ (YSI, Yellow Springs, OH, USA). A portable pH/DO meter (Thermo Fisher Scientific, Inc., Beverly, MA, USA) measured the dissolved oxygen (DO) concentrations and pH values. The sampling collection was performed at the low tide of about 50~100 cm. Ten (for *T.* *hemprichii*, *Cymodocea* *nodos* and *Syringodium* *isoetifolium*) or thirty (for *H. ovalis,* small plant size) seagrass shoots containing leaves, rhizomes, and roots were collected manually with great caution to avoid the damages, and distance between them was about one meter. After the seagrass plants were separated from the substrate, the loosely attached sediment was gently shaken from the roots manually with approximately 1 mm of sediment still attached to the roots. Samples for microbial and physicochemical analysis were immediately put in an icebox (4 °C) in the dark and then transported to the laboratory or the cruise ship within three hours. Samples collected in Daya Bay were preprocessed in Daya Bay Marine Biology Research Station, Sanya Bay in Tropical Marine Biological Research Station in Hainan, the Xisha Islands in Xisha Deep Sea Marine Environment Observation and Research Station, and Nansha island on the survey vessel. The seagrass roots were placed in a 50 mL sterile conical tube containing 25 mL phosphate-buffered saline (PBS) solution and vortexed for 30 s. The obtained turbid solution was filtered through a 100-μm nylon mesh, and then the filtrate was centrifuged to obtain the seagrass rhizosphere sediment [28,29], then the samples were stored at −80 °C in the lab or lipid nitrogen until DNA extraction. Inorganic nutrients in seawater, including ammonium, nitrate, nitrite, and phosphate, and total phosphorus (TP) in sediment were measured in triplicate according to the standard oceanography methods (General Administration of Quality Supervision, Inspection, and Quarantine of the People’s Republic of China, 2002). Total carbon (TC), total organic carbon (TOC), and total nitrogen (TN) were measured by CHNS Vario E1 III elemental analyzer (Hanau, Germany), according to Sun et al. (2020) [30].

### 2.3. DNA Extraction, PCR, Library Preparation, and Illumina MiSeq Sequencing

Microbial DNA from approximately 1 g of rhizosphere sample was extracted with EZNA^®^ Soil DNA kit (Omega Bio-Tek, Norcross, GA, USA), quantified by Qubit 2.0 fluorometer (Invitrogen, Carlsbad, CA, USA), and checked on 1% agarose gel. The 16S rDNA sequencing library preparations and sequencing were conducted at GENEWIZ, Inc. (Suzhou, China). Thirty nanograms of total DNA was used to amplify the target amplicons with a MetaVx^TM^ library preparation kit (Genewiz, Inc., South Plainfield, NJ, USA). The forward primers sequence “CCTACGGRRBGCASCAGKVRVGAAT” and reverse primers “GGACTACNVGGGTWTCTAATCC” were used to amplify the V3-V4 region of 16S rDNA [31]. Two primer pairs were used to amplify more microbes, and then more variations of the microbes were identified in the samples. The PCR mixture contained 20 ng DNA template, 2.5 µL 10× TransStart Buffer, 0.5 µL 2.5 U/µL TransStart Taq, 2.0 µL dNTPs (2.5 mM each), an d2.5 µL 1× primers mix. Distilled H_2_O was used as the template for negative controls. The PCR protocol consisted of an initial 3-min denaturation at 94 °C, followed by 14 cycles of denaturing at 94 °C for 50 s, annealing at 57 °C for 90 s, extension at 72 °C for 10 s, and completed with a final extension at 72 °C for 5 min. The libraries for downstream NGS sequencing were constructed according to Li et al. (2017) [31], and then were loaded and sequenced on an Illumina MiSeq instrument according to the manufacturer’s instructions (Illumina, San Diego, CA, USA). Sequencing was performed using a 2 × 300/250 paired-end (PE) configuration. All the raw sequences obtained from this study were deposited in the NCBI sequence read archive (SRA) under accession number PRJNA497291.

### 2.4. Statistical Analysis 

The resultant amplicon sequencing, bioinformatics, was mainly performed in R package EasyAmplicon v1.0 according to Liu et al. [32]. The raw amplicon paired-end reads were grouped based on their barcode sequences and then were merged to obtain amplicon sequences. The joined sequences were then quality-filtered by “usearch10 –fastq_filter –fastq_maxee_rate 0.01”, and the barcode and primers sequences were removed during this process. Criteria for the quality filtering were as following: sequence length >200 bp, ambiguous bases, mean quality score >20. All representative sequences were grouped into operational taxonomic units (OTUs) using the clustering program USEARCH (https://www.drive5.com/usearch/ (accessed on 17 August 2020)) against the Silva 138 Reference database [33]. The singletons and chimeric sequences were also removed. The obtained representative sequences of each OTU were used to construct the phylogenetic tree by FastTree after aligned by sequence alignment program Multiple Alignment using Fast Fourier Transform (MAFFT) 7.0 [34,35,36]. 

All OTUs identified as belonging to the chloroplast, mitochondria, Archaea, and Eukaryote were removed from the data set before downstream analyses. The remaining sequences were rarefied before calculating alpha diversity statistics analysis, and all statistical analyses were conducted in the open source statistical language R (R Core Team, 2018, version 3.4.4). The alpha diversity (observed OTU richness, the Shannon index, Simpson index, and phylogenetic diversity (PD) were calculated in R (“vegan”). Venn diagram plot shared OTUs, and Unique OTUs were generated by the R package “VennDiagram” [37]. 

Non-parametric tests (999 permutations) of the multiple-response permutation procedure (MRPP) were performed based on weighted UniFrac distance, Bray-Curtis distance, Euclidean distance, and Sorensen distance to measure the dissimilarity of microbial communities, respectively [38,39]. Gene functional annotation based on all obtained sequencing was performed against the FAPROTAX dataset; the dataset and associated software are available at http://www.loucalab.com/archive/FAPROTAX/ (accessed on 17 August 2020) [40]. FAPROTAX dataset is a manually constructed database that maps prokaryotic taxa (e.g., genera or species) to putative functions based on the literature on cultured representatives [40]. The core microbial community in this study was defined as those present in all samples, including all three replicates [11], and their network analysis was further analyzed based on a random matrix theory (RMT)-based method according to Deng [41] and Luo [42]. Network analysis of the core microbial community was processed using by molecular Ecological Network Analysis Pipeline (http://ieg4.rccc.ou.edu/MENA/ (accessed on 17 August 2020)) and visualized in an open-source software platform Cytoscape (v.3.6.0). 

## 3. Results

### 3.1. Sampling Locations and Physicochemical Properties of the Seagrass Rhizosphere 

The sampling location and all the measured physicochemical parameters of investigated samples are shown in Table 1. All four seagrass species across four coral reef ecosystems were studied. There were variations in the physicochemical parameters for different samples. For instance, pH values varied between 8.14 and 8.24 ± 0.03. The salinity range was from 24.70 ± 0.3 to 34.57 ± 0.08 practical salinity unit (PSU), with the lowest value detected at SYB. The highest concentration of nitrate was recorded in the region DYB as 0.153 ± 0.004 mg/L, while SYB had the highest concentration of ammonium (0.121 ± 0.002 mg/L). The range of the DO was from 5.82 ± 0.05 to 9.64 ± 0.59 mg/L. The highest TN, TC, and TOC concentration existed in the sediment of XSC. 

### 3.2. Taxonomy, Phylogenetic Diversity, and Composition of Rhizosphere Bacterial Communities of the Seagrass 

**Figure 2:**
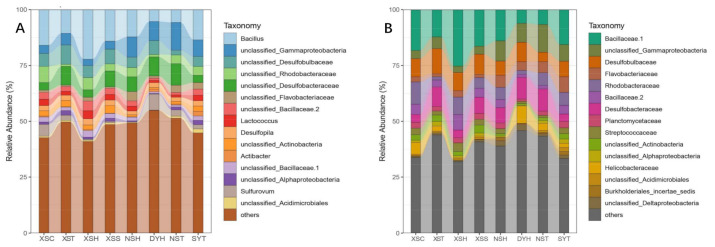
The relative abundance of the microbial composition of seagrass rhizosphere samples across four coral reef ecosystems at the family (**A**) and genus (**B**) level, respectively (DYH indicates seagrass *Halophila ovalis* from Daya Bay; SYT indicates seagrass *Thalassia* *hemprichii* from Sanya Bay, XST, XSH, XSC, and XSS indicate *T.* *hemprichii*, *H. ovalis*, *Cymodocea* *nodos* and *Syringodium* *isoetifolium* collected from the Xisha Islands, and NSH and NST indicates seagrass *H. ovalis* and *T.* *hemprichii* collected from the Nansha Islands).

Total sequences assigned to all samples were 1,601,000. After removing chimeric and singleton, the remaining sequences were 1,332,470 belonging to 2423 OTUs, and the minimum read of all samples was 33,987. Then, sixteen OTUs related to chloroplast/mitochondria were removed. The resampling depth was 33,980 merged sequences, with 2405 OTUs left after resampling. All OTUs were grouped into 33 phyla and 290 genera. The rarefaction curves showed that the sequencing depth was relatively enough to cover bacterial diversity as all the rarefaction curves almost reached saturation plateaus (Figure S1), and the minimum coverage of the samples was 98.96% (Table S1). The phylum Proteobacteria accounted for 33.47% of bacterial communities, followed by phylum Bacteroidetes (23.33%) and Planctomycetes (12.47%). At the genus level, the dominant genera were *Bacillus* (phylum Firmicutes), and the range of its relative abundance for each sample was from 4.61% to 24.04%. The highest abundance was detected in sample XSH (the Xisha Islands), while the lowest sample was DYH (Daya Bay). The other dominant genera were unclassified Desulfobulbaceae (class Deltaproteobacteria), unclassified Rhodobacteraceae (class Alphaproteobacteria), unclassified Bacillaceae 2 (phylum Firmicutes), Lactococcus (phylum Firmicutes), and unclassified Desulfobacteraceae (class Deltaproteobacteria and Desulfopila (class Deltaproteobacteria) (Figure 2).

The alpha diversity, including PD, Chao1, Richness (Observed OTUs), Shannon, and Simpson index of all samples, was calculated, and is shown in Figure S2. The highest Faith’s PD value was detected in the samples of NST with a value of 80.92 ± 7.95, while the lowest Faith’s PD was 59.3 ± 5.13 for the samples of DYH. Meanwhile, the highest value of Chao1, Richness, Shannon, and Simpson index also existed in the samples of NST. Based on seagrass species (*C. nodosa*, *T. hemprichii*, *H. ovalis* and *S. isoetifolium*) collected from XS, the alpha index of PD, Richness, Shannon, and Simpson demonstrated significant differences with the *p-*value below 0.05 (Table 2). For different sampling locations, seagrass *H. ovalis* from Daya Bay, the Xisha Islands, and the Nansha Islands showed significant variations in Richness, Shannon, and Simpson (*p* < 0.05), while for seagrass *T. hemprichii* from Sanya Bay, the Xisha Islands and the Nansha Islands exhibited significant in Richness and Shannon (*p* < 0.05). The beta diversity analyzed by MRPP based on bray-cutis dissimilarity, Euclidean distance, and Sorensen distance demonstrated that there were significant variations (*p* < 0.05) for four seagrass species from the same location, while no significant variations were detected for seagrass *T. hemprichii* and *H. ovalis* from different sampling locations (*p* > 0.05) (Table 3).

### 3.3. Potential Functional Roles of Microbial Played in Seagrass Rhizosphere

**Figure 3:**
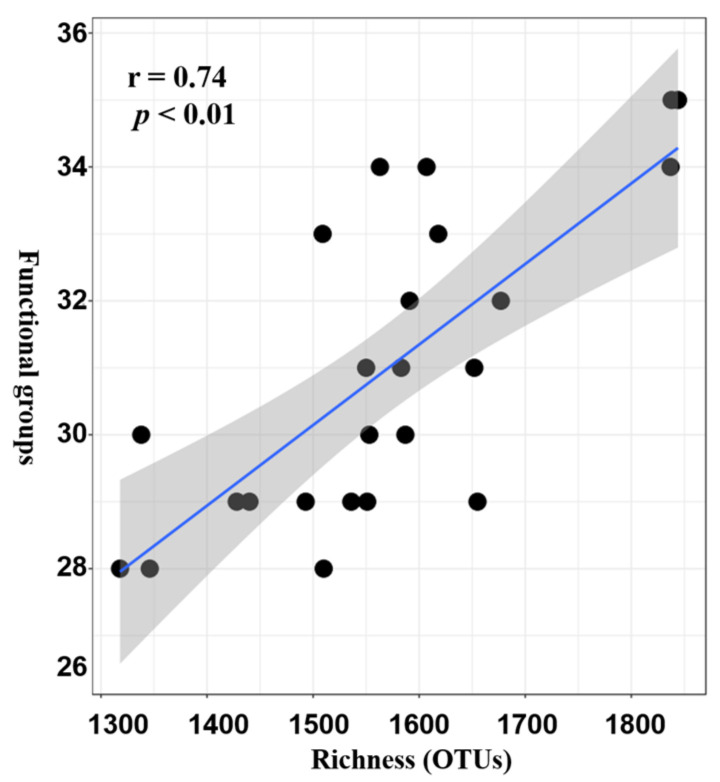
Spearman’s correlation of community species diversity (richness) and functional diversity of all functional groups.

The FAPROTAX database comprises 7820 members (4724 unique members) belonging to 90 groups. The results of this study showed that there were 920 assignment records to groups, and 427 out of 2405 records (17.75%) were assigned to at least one group. In total, 36 functional groups were represented (i.e., associated with at least one record). As illustrated in Figure S3, the top four dominant functional groups were sulfate respiration (129 records), respiration of sulfur compounds (131 records), aerobic chemoheterotrophy (192 records), and chemoheterotrophy (247 records), respectively. The ranges of relative abundance of these four groups in all the samples were 11.75–21.44%, 11.75–21.48%, 7.72–11.34%, and 3.29–8.20%. Further analysis revealed that the sulfate respiration group mainly consists of nine genera, including *Desulfatiglans*, *Desulfobulbus*, *Desulfocarbo*, *Desulfomonile*, *Desulfopila*, *Desulfosarcina*, *Desulfovibrio*, unclassified *Desulfobacteraceae,* and unclassified *Desulfobulbacea*, while for the respiration of sulfur compounds, 11 genera had participated in this process. The diversity of microbes that participated in the procedure of chemoheterotrophy and aerobic chemoheterotrophy is very high. Ninety and sixty-eight genera of microbes were identified, respectively. Spearman’s correlation was employed to test the relationship between alpha diversity (richness) and functional groups obtained, and results showed that there was a positive correlation (r = 0.74, *p* < 0.01) (Figure 3). 

### 3.4. Venn Diagram Analysis of the Variations in Taxonomy Species and Functional Groups

**Figure 4:**
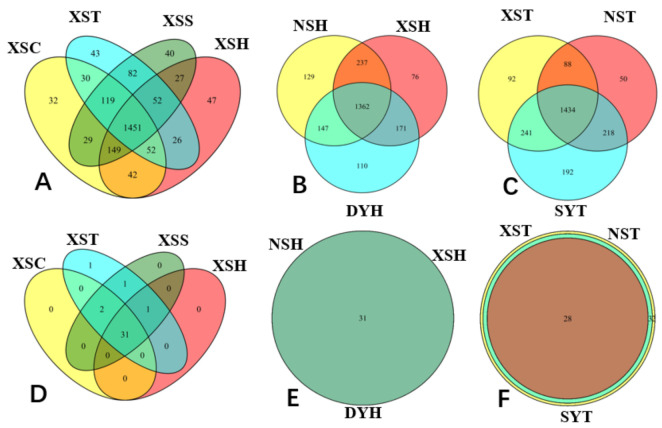
Venn diagrams analysis of the microbial OTUs and putative functional groups. Venn diagrams showing the unique and shared OTUs numbers (**A**) between four seagrass species in the Xisha Islands; (**B**) three sampling locations of seagrass *H. ovalis*; (**C**) three sampling locations of seagrass *T. hemprichii*. Venn diagram showing the unique and shared functional groups (**D**) between four seagrass species in the Xisha Islands; (**E**) three sampling locations of seagrass *H. ovalis*; (**F**) three sampling locations of seagrass *T. hemprichii.* DYH indicates seagrass *H. ovalis* from Daya Bay; SYT indicates seagrass *Thalassia hemprichii* from Sanya Bay, XST, XSH, XSC, and XSS indicate *T. hemprichii*, *H. ovalis*, *Cymodocea nodos* and *Syringodium isoetifolium* collected from the Nansha Islands.

Based on the seagrass species, the OTUs shared by four seagrass species collected in XS were 1451, and each species had its unique OTUs (Figure 4). Among them, sample XSH had the highest unique OTUs with 47, followed by XSC. Meanwhile, seagrass *H. ovalis* shared 1362 OTUs with the same species from three sampling locations based on the sampling location. Moreover, for seagrass *T. hemprichii*, the shared OTUs were 1434, with sample NST possessing 192 unique OTUs. As for the functional structure, from the seagrass species perspective, the functional groups they shared were 31, and no unique functional groups were detected. XST had 33 functional groups and followed by NST having 31 functional groups. Moreover, for seagrass *H. ovalis*, samples from three sampling locations shared all their functional groups (31), while for seagrass *T. hemprichii*, they shared 28 functional groups. Furthermore, for seagrass *T. hemprichii*, the lowest number of functional groups is 28 detected in seagrass SYT, and the highest value is 33 from samples XST (Figure 4).

Variations in seagrass rhizosphere microbial communities at taxonomical and functional levels were analyzed based on species and locations, respectively. The top abundant 50 genera were included for further taxonomical structure analysis (Tables S2–S4), and all detected functional groups (36) were included for analysis (Tables S5–S7). Most of the investigated genera demonstrated significant differences between different species based on seagrass species (Table S2). Several genera for species’ comparison between two seagrass species from the Xisha Islands, such as Desulfopila, unclassified Bacteroidales, and Eudoraea, which showed no significant differences (*p* > 0.05). For site-based analysis, many of those genera exhibited substantial variations among the sampling sites, such as genus unclassified Desulfobulbaceae and unclassified Chloroflexi. In contrast, *Desulfopila***,** *Oceanobacillus*, unclassified Syntrophobacterales, *Desulfobulbus* of seagrass *T. hemprichii* (*p* < 0.05), and *Desulfosarcina* of seagrass *H. ovalis* showed no significant differences (*p* > 0.05).

All investigated samples shared many of the functional groups, but significant differences were also detected in the samples at both species-based and location-based levels. For instance, methanogenesis’s functional groups, by reducing methyl compounds with H_2_, Hydrogenotrophic methanogenesis, and methanogenesis, could be found in all the seagrass species samples collected from the Xisha Islands. However, none of the analyzed genera and detected functional groups showed both species differences and location differences. 

### 3.5. Core Microbial Community in Seagrass Microbial Rhizosphere

**Figure 5:**
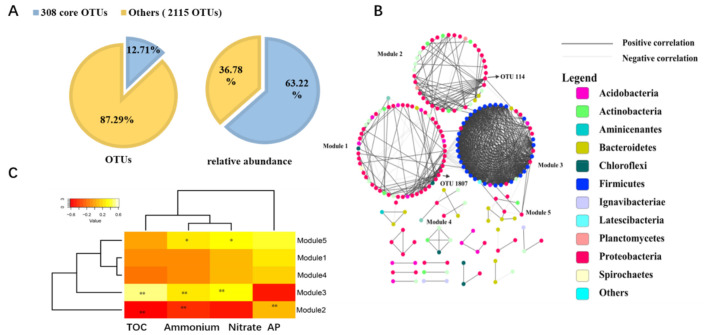
The core community composition and its network analysis of the core microbial community of all seagrass rhizosphere microbial communities. (**A**) the core OTUs number and its relative abundance; (**B**) Modules (groups of OTUs) and only module more than five OTUs are shown with module numbers. The colored circles indicate those OTUs affiliated with phyla (the color code’s legend on the right). OTU114 and OTU1807 are module hubs. Black links represent positive correlations, and grey links represent negative correlations; (**C**) The correlations between five modules eigengenes and environmental factors (* indicates a statistically significant correlation: * *p* < 0.05, and ** *p* < 0.01).

The co-occurrence network method was used to explore the interaction between the rhizosphere microbes and to identify the keystone species. In all, 308 of 2405 OTUs were identified as core OTUs shared by all samples. They accounted for 12.81% of all obtained OTUs, and their relative abundance was 61.83% (Figure 5A). The core OTUs belonged to 14 phyla and 89 genera, and the predominant phyla were Proteobacteria (24.37%), Firmicutes (21.03%), and Bacteroidetes (3.37%). Afterward, 197 OTUs were selected for network analysis, and the correlation network was generated with a coefficient cutoff of 0.760, as determined by the RMT-based algorithm. There was a total of 773 edges (136 negative correlations and 637 positive correlations), and most of the correlation was positive (82.41%) (Figure 5B). In all, 19 modules were constructed, and the biggest module was Module 1, consisting of 58 OTUs, followed by Module 3 with 44 OTUs. The OTU 114 and OTU 1807 (phylum Proteobacteria) were identified as module hubs (OTU highly connected in the own module) in Figure S4. Modules with more than five OTUs were included for correlation analysis of module eigengenes and environmental factors. The modules’ responses to the environment were different, and Figure 5C showed that the measured physiochemical factors were significantly correlated with module eigengenes of Module 2, 3, and 5. TOC, ammonium, and nitrate were negatively correlated with Module 2 while positively correlated with Module 3. However, Module 1 and 4 were not significantly correlated with the environmental parameters. 

## 4. Discussion

### 4.1. Variations in the Taxonomical, Phylogenetical Diversity and Composition of Bacterial Communities

Significant variations in PD and taxonomical diversity of bacterial communities could be detected based on seagrass species within a coral reef ecosystem, but only significant variations in taxonomical diversity for the same seagrass species from different sampling locations (Table 2). Moreover, significant taxonomical and phylogenetic variations only existed among different seagrass species collected from the Xisha Islands. Therefore, the coral reef ecosystem’s seagrass species may be one important factor in shaping the rhizosphere bacterial communities.

We also found significant differences in the taxonomy composition of rhizosphere bacterial communities at the genus level based on different seagrass sampling locations. This was partly consistent with the investigation result of Cúcio et al. (2016) [16], the result of which demonstrated that significant differences were detected for the same seagrass species from different sampling locations, but no significant differences existed between the rhizobiomes of different seagrass species from the same sampling location. The reason for this phenomenon may be that different seagrass species were included in each study. Three different seagrass species, namely *Z. marina*, *Z. noltii*, and *Cymodocea nodos*, were studied for Cúcio et al. (2016) [16], while four seagrass species (*C. nodos*, *T. hemprichii*, *H. ovalis,* and *S. isoetifolium*) were examined in our investigation. Another reason for this discrepancy may be the different growth habits. The seagrass habitats for their study was in the intertidal regions, while all the seagrasses in this study were collected from the coral reef ecosystem [16]. Moreover, previous studies have highlighted the importance of temperature in constructing the rhizosphere bacterial community anwhich exhibited seasonal variations [43,44]. Therefore, there may also be seasonal variations in the seagrass rhizosphere bacterial community. More investigation on the temporal scale in the future needs to be performed.

Proteobacteria (class alpha-, beta-, delta-, gamma-, and epsilon-proteobacteria) and the Firmicutes were the two most predominant phyla across the four coral reef ecosystems. Besides, class Deltaproteobacteria accounted for over 20% of all investigated bacterial communities. Cúcio et al. (2016) also reported that the phylum Proteobacteria was the most dominant in the rhizomes of seagrass *Z. marina*, *Z. noltii*, and *Cymodocea nodosa*, with the proportion ranging from 65% to 68%. The existence of plants played a crucial role in shaping the microbial community in the rhizosphere of seagrasses as the seagrass rhizosphere bacterial community composition was quite different from that of the surrounding water and bulk sediment [16]. Besides, seagrass (*Z. marina*) colonization increased the abundance of the nitrogen fixation bacteria and other bacteria involved in benthic carbon and sulfur cycling [45]. 

Moreover, some OTUs were peculiar to one coral reef ecosystem, and each coral reef had its own individual OTUs in our study. For instance, OTU1109 was affiliated to class Phycisphaerae SHA-43 belonging to the phylum Planctomycetes and could only be discovered at XS. It may play an important role in the nitrogen cycle by participating in the anammox process, which was assumed as a predominant source of N_2_ production in anoxic marine environments [46,47,48]. Moreover, bacteria from the family *Rhodothermaceae* (phylum Bacteroidetes) were specially retrieved from Sanya Bay, and microorganisms from this family were usually isolated from the extreme environments and exhibited extreme thermophilic or halophilic characteristics [49,50]. 

### 4.2. The Functional Structure of Microbial Communities in Seagrass Rhizosphere 

Seagrass holobionts have been reported to play essential roles in the cycle of sulfur, nitrogen, and carbon, at both microbial structural and functional levels [11,13], and Ugarelli et al. (2019) [51] reported that the seagrass plant and its microbiome were highly interlinked in the cycle of sulfur, nitrogen, and carbon. Likewise, FAPROTAX analysis revealed that many microbes in the seagrass rhizosphere of coral reef ecosystems participated in these processes (Figure S3). 

Previous studies showed that increased sulfide concentration in the sediment caused by the activity of sulfate-reducing prokaryotes was one of the main reasons for seagrass death all over the world [11,13]. However, seagrass could oxygenate their roots [41] and lose radial oxygen in the rhizosphere of young roots to lower the concentration of sulfide to protect themselves [52]. What is more, sulfur-oxidizing bacteria in this ecosystem may also alleviate the sulfide stress for seagrass by oxidation of sulfide [53]. A higher abundance of genes was found to participate in the process of sulfur oxidation than sulfate reduction in the rhizosphere of the seagrass *Z. marina* [14]. We also found a high percentage of sulfate respiration (129 records) and respiration of sulfur compounds (131 records) in the FAPROTAX analysis result. This may indicate that microbes in the seagrass rhizosphere also play an important role in the sulfur-related cycle in the coral reef ecosystem. 

Bioavailable nitrogen is crucial to all living organisms, but it is still a limiting nutrient globally [54]. The nitrogen enters the ecosystem from the air in the form of ammonia by the microbial nitrogen fixation, which is an essential link of the nitrogen cycle due to nitrogen usually acting as the limiting factor for productivity in the oligotrophic seagrass meadow and coral reef ecosystems [55]. Welsh et al. (2002) found that the microbes capable of sulfate-reducing are the significant component of the diazotrophs in many seagrass ecosystems [56]. Besides, the microbes involved in the nitrogen cycle, as revealed by FAPROTAX analysis in this study, mainly involved in the process of nitrification, aerobic ammonia oxidation, nitrate reduction, nitrate respiration, and nitrogen respiration. 

Furthermore, the microbes conducted of nitrification activity mainly came from the genus *Nitrosopumilus*, *Nitrososphaera,* and *Nitrospira*. Nitrification is a process of oxidizing ammonia via nitrite to nitrate, which was assumed as a two-step process catalyzed by chemolithoautotrophic microorganisms before 2015 [57,58]. Daims et al. (2015) have reported that a completely nitrifying bacterium from the genus *Nitrospira*, which was present in diverse environments, and those findings confirmed that completely nitrifying *Nitrospira* played important roles in the nitrogen cycle-related microbial functional groups [58]. Although the ammonia available concentrations in most ocean waters are low, this is suitable for the living of comammox organisms. However, no comammox gene has been found in ocean waters until now. To explore microbes capable of comammox, a future research hotspot for environmental microbiologists is underway [54]. 

The diversity of carbon metabolism found in this study was very high, such as aerobic chemoheterotrophy, chemoheterotrophy, fermentation, aromatic compound degradation, photoautotrophy, methanogenesis, and methylotrophy (Figure S3). Many microbes of the phylum Planctomycetes were involved in the process of aerobic chemoheterotrophy. Like the genus *Blastopirellula,* a dominant chemoorganotrophic genus in the Black Sea sediments, are chemoheterotrophic [59,60], and their the major carbon and energy sources are carbohydrates [59]. Eight OTUs were detected in the methylotrophy from the genus *Methanomassiliicoccus*, unclassified Methylophilaceae, and *Methylophaga*, which accounted for 0.87% of all detected functional groups. Moreover, the putative methylotrophic bacteria, such as *Methylotenera* and *Methylophaga,* were more abundant in healthy seagrasses and could be used as indicators of seagrass health root microbiomes [61]. Besides, the microbes involved in sulfur-cycling, including sulfide-oxidizing (e.g., Candidatus Thiodiazotropha and *Candidatus Electrothrix*) and sulfate-reducing (e.g., SEEP-SRB1, *Desulfomonile*, and *Desulfonema*), were more abundant in stressed seagrass [61]. Hence, there is a need to investigate the relationship between the composition and functions of rhizosphere microbes and seagrass health. 

### 4.3. The Core Microbial Community in Seagrass Rhizosphere across the Four Coral Reef Ecosystems

Identification of the core microbial community may provide the cues for understanding the key players in sustaining the growth and health of the seagrass, regardless of the seagrass species and locations. The taxonomy of the predominant core microbial community in this study was Desulfobulbaceae (phylum Proteobacteria), Bacillaceae 1 (phylum Firmicutes), Rhodobacteraceae (phylum Proteobacteria), and Streptococcaceae (phylum Firmicutes). While for seagrass *Z. marina*, *Z. noltii,* and *Cymodocea nodosa* [16], the core seagrass rhizobiome consisted of 0.2% of all OTUs, about 12.81% of all obtained OTUs were identified as core OTUs for the sample investigated in this study. The core microbial communities of different seagrass species and distributing locations may have different community composition and species specialty. The effect of the different environmental factors in the different sampling sites could one of the reasons contributing to this phenomenon [51]. 

Most of the bacterial core community in the seagrasses rhizosphere was involved in the sulfur cycle [16]. The FAPROTAX analysis from our investigation of the sulfur-related biogeochemical cycle showed that sulfate respiration, dark oxidation of sulfur compounds, and sulfite respiration were dominant in all putative functional groups. For instance, the FAPROTAX analysis showed that genus *Desulfocarbo*, *Desulfatiglans*, *Desulfosarcina*, *Desulfobulbus*, *Desulfopila*, *Desulfovibrio*, *Desulfomonile*, unclassified Desulfobulbaceae, *Desulfovibrio*, *Desulfomonile*, and unclassified Desulfobacteraceae participated in the sulfate respiration process, and 18.76% of the core community was affiliated to above-related genera. Network analysis demonstrated that two module hubs were OTU114 and OTU1807, and they were affiliated to family Rhodobacterales and class Gammaproteobacteria, respectively. Family Rhodobacterales was predicted to play an important role in the process of aerobic chemoheterotrophy and chemoheterotrophy, thus they were involved in the carbon cycle of the seagrass ecosystem. Compared to the neighboring bulk sediment, the bacterial production in the seagrass rhizosphere exhibited a diel pattern that the production rates could be two times higher during daytime than at night [62,63]. In the oligotrophic seagrass meadow, DOC excreted from the seagrass rhizosphere accounted for a large proportion of carbon source for sediment bacteria [64].

In this study, the microbes’ carbon cycle was mainly involved in chemoheterotrophy, aerobic chemoheterotrophy, phototrophy, and fermentation. 

## 5. Conclusions

This investigation mainly focused on the taxonomy structure and functional variations, and core microbial community of seagrass rhizosphere across four coral reef ecosystems. The predominant microbial phyla were Proteobacteria, Bacteroidetes, and Planctomycetes, and the four major functional groups were sulfate respiration, respiration of sulfur compounds, aerobic chemoheterotrophy, and chemoheterotrophy in this study. In the aspect of alpha diversity, significant differences existed based on seagrass species and sampling locations, while no such variations were detected for beta diversity between different sampling locations. The investigated seagrass rhizosphere microbial communities demonstrated significant community composition variations at the genus level, and functional groups also differed among different seagrass species (four seagrass species in the Xisha Islands) and the same seagrass species (seagrass *H. ovalis* and *T. hemprichii*) from different locations. The core microbial community of all microbial communities was identified, and most of the microbes had a positive correlation (82.41%). Two module hubs, OTU114 and OTU1807, affiliated to family Rhodobacterales and class Gammaproteobacteria, respectively, were identified as the keystone species. In addition, TOC, ammonium, and nitrate were the significant environmental factors correlated with the core microbial community structure. This study will provide new insight into the seagrass rhizosphere microbiome of coral reef ecosystems and will contribute to more effective management for ecological conservation and restoration policymaking for coral reef ecosystems, such as supplying the scientific data for directed isolation of functional bacteria belonging to the core microbe or species-specific seagrass rhizosphere, thus allowing for characterization of the isolated strain’s functional ability and measurement of their seagrass-promoting traits by bacteria inoculant. 

## Figures and Tables

**Table 1:** Typical environmental factors of the collected samples (the standard errors in parentheses).

Sample		DYH	SYT	XST	XSH	XSC	XSS	NXH	NXT
Latitude (° E)		22.54	18.2	16.84	16.84	16.84	16.84	9.71	10.22
Longtitude (° N)		114.45	119.47	112.34	112.34	112.34	112.34	114.29	114.2
Water	pH	8.14 (0)	8.15 (0.01)	8.24 (0.01)	8.24 (0.03)	8.24 (0.03)	8.24 (0.01)	8.15 (0.01)	8.21 (0.01)
	Salinity	28.37 (0.07)	24.7 (0.3)	34.52 (0.05)	34.52 (0.15)	34.57 (0.08)	34.52 (0.05)	34.31 (0)	34.54 (0.09)
	DO (mg/L)	5.82 (0.05)	6.7 (0.2)	9.47 (0.17)	9.47 (0.51)	9.64 (0.59)	9.47 (0.17)	7.64 (0.03)	8.36 (0.13)
	Nitrate (mg/L)	0.153 (0.004)	0.044 (0.002)	0.049 (0.002)	0.049 (0.005)	0.046 (0.001)	0.049 (0.002)	0.06 (0.001)	0.035 (0)
	Nitrite (mg/L)	0.001 (0)	0.025 (0.001)	0.016 (0)	0.016 (0.001)	0.016 (0.001)	0.016 (0)	0.001 (0)	0.001 (0)
	Ammonium (mg/L)	0.069 (0)	0.121 (0.002)	0.094 (0.002)	0.094 (0.005)	0.091 (0.006)	0.094 (0.002)	0.048 (0.001)	0.032 (0.001)
	Phosphate (mg/L)	0.007 (0.001)	0.015 (0)	0.01 (0.001)	0.01 (0.002)	0.01 (0.001)	0.01 (0.001)	0.006 (0)	0.006 (0)
Sediment	Ammonium (mg/kg)	3.02 (0.01)	5.97 (0.02)	4.37 (0.01)	2.91 (0.03)	1.45 (0.01)	4.78 (0.02)	6.39 (0.03)	5.48 (0.12)
	Nitrate (mg/kg)	22.3 (2.63)	24.4 (3.68)	44.0 (2.10)	62.4 (3.26)	69.4 (4.06)	44.0 (2.24)	32.60 (1.12)	45.60 (1.56)
	AP (mg/kg)	18.0 (2.01)	15.0 (1.28)	14.0 (3.06)	11.0 (1.85)	15.0 (2.30)	15.0 (1.60)	18 (2.45)	17 (3.01)
	TOC (%)	18.4% (1.89)	32.5% (2.36)	43.4% (1.03)	33.2% (2.64)	40.3% (3.12)	21.8% (3.56)	36.80 (2.63)	42.06 (3.42)

Note: DYH indicates seagrass *Halophila ovalis* from Daya Bay; SYT indicates seagrass *Thalassia* *hemprichii* from Sanya Bay, XST, XSH, XSC, and XSS indicate *T.* *hemprichii*, *H. ovalis*, *Cymodocea* *nodos* and *Syringodium* *isoetifolium* collected from the Xisha Islands, and NSH and NST indicates seagrass *H. ovalis* and *T.* *hemprichii* collected from the Nansha Islands.

**Table 2:** The comparison analysis of phylogenetic and taxonomy alpha diversity based on different seagrass species of same sampling location and the same seagrass species of different sampling locations, respectively.

	Microbial Communities	Microbial Communities	Phylogenetic Composition (PD)	Taxonomic Alpha Diversity		
				Richness	Shannon	Simpson
Species			*p*	*p*	*p*	*p*
	XSC	XST	0.9420	0.6700	0.5250	0.3240
	XSC	XSH	0.4550	0.3870	0.7380	0.7380
	XSC	XSS	0.5250	0.6700	0.3240	0.5250
	XST	XSH	0.0802	0.9690	0.0810	*0.0330*
	XST	XSS	0.2180	0.1060	0.9870	0.9870
	XSS	XSH	*0.0240*	*0.0330*	*0.0330*	0.0810
Location						
*H. ovalis*	XSH	DYH	0.9500	0.3700	*0.0200*	*0.0200*
	XSH	CGX	0.2300	0.3700	0.3700	0.3700
	DYH	CGX	0.1300	*0.0200*	0.3700	0.3700
*T. hemprichii*						
	XST	SYT	0.5490	0.5500	0.3700	0.8960
	XST	NXT	0.5490	0.3000	0.0370	0.0650
	SYT	NXT	0.0930	*0.0300*	*0.0200*	0.1730

Note: DYH indicates seagrass *Halophila ovalis* from Daya Bay; SYT indicates seagrass *Thalassia hemprichii* from Sanya Bay, XST, XSH, XSC, and XSS indicate *T. hemprichii*, *H. ovalis*, *Cymodocea nodos* and *Syringodium isoetifolium* collected from the Xisha Islands, and NSH and NST indicates seagrass *H. ovalis* and *T.* *hemprichii* collected from the Nansha Islands; The *p* value lower than 0.05 (threshold for significance) is shown in italic.

**Table 3:** The comparison analysis of beta diversity based on seagrass species and sampling locations, respectively.

	Microbial Community	Microbial Community	Delta Unifrac	*P* Unifrac	Delta Bray	*P* Bray	Delta Euclidean	*P* Euclidean	Delta Sorensen	*P* Sorensen
**Species**										
	XSC	XST	0.184	0.087	0.294	0.016	1.260	0.156	0.099	0.293
	XSC	XSH	0.177	0.084	0.308	0.109	1.590	0.294	0.152	0.282
	XSC	XSS	0.049	0.100	0.088	0.100	1.040	0.500	0.061	0.300
	XST	XSH	0.219	*0.025* *	0.366	*0.015* *	1.429	*0.017* *	0.136	0.124
	XST	XSS	0.176	*0.035* *	0.284	*0.017* *	1.073	*0.009* **	0.083	*0.016* *
	XSH	XSS	0.169	*0.011* *	0.299	*0.012* *	1.403	*0.037* *	0.135	0.109
**Location**										
***H. ovalis***	XSH	NSH	0.039	0.100	0.088	0.100	1.024	0.100	0.111	0.100
	XSH	DYH	0.035	0.100	0.125	0.100	1.024	0.100	0.114	0.100
	NSH	DYH	0.033	0.100	0.122	0.100	0.667	0.100	0.042	0.100
***T. hemprichii***	XST	NST	0.070	0.100	0.110	0.100	20.104	0.100	0.130	0.100
	XST	SYT	0.064	0.100	0.112	0.100	19.395	0.100	0.132	0.100
	NST	SYT	0.026	0.100	0.105	0.100	18.762	0.100	0.121	0.100

Note: (Values values in the table represent *p* values (* *p* < 0.05, ** *p* < 0.01) (DYH indicates seagrass *Halophila ovalis* from Daya Bay; SYT indicates seagrass *Thalassia hemprichii* from Sanya Bay, XST, XSH, XSC, and XSS indicate *T. hemprichii*, *H. ovalis*, *Cymodocea nodos* and *Syringodium isoetifolium* collected from the Nansha Islands) (The *p* value lower than 0.05 (threshold for significance) is shown in italic).

## Data Availability

All the raw sequences obtained from this study were deposited in the NCBI sequence read archive (SRA) under accession number PRJNA497291.

## Supplementary Materials

The following are available online at https://www.mdpi.com/article/10.3390/life11080852/s1, Figure S1: Rarefaction curves of OUT diversity in each sample; Figure S2. Phylogenetic and taxonomic alpha diversity of seagrass rhizosphere microbial communities (A: phylogenetic diversity; B: richness; C: Shannon; D: Simpson); Figure S3. The bubble plot of functional groups in all rhizosphere microbial communities of seagrass; Figure S4. Zi-Pi plot of network nodes of core microbial community shared by all samples; Table S1. The sequences characteristics of the all obtained seagrass rhizosphere samples; Table S2. The significance of differences in taxonomy structure of microbial community between different seagrass species (A) and seagrass *T. hemprichii* (B) and *H. ovalis* (C) from different sampling sites on the genus level; Table S3. The significance of differences in taxonomy structure of microbial community between seagrass *H. ovalis* from different sampling sites on the genus level; Table S4. The significance of differences in taxonomy structure of microbial community between seagrass *T.* *hemprichii* from different sampling sites on the genus level; Table S5. The significance of differences in functional groups of microbial community between different seagrass species on the genus level; Table S6. The significance of differences in functional groups of microbial community between seagrass *T.hemprichii* from different sampling sites on the genus level; Table S7. The significance of differences in functional groups of microbial community between different seagrass *H.ovalis* from different sampling sites on the genus level.

## References

[1] Hughes T.P., Barnes M.L., Bellwood D.R., Cinner J.E., Cumming G.S., Jackson J.B.C., Kleypas J., van de Leemput I.A., Lough J.M., Morrison T.H. (2017). Coral reefs in the Anthropocene. Nature.

[2] Andersson A.J., Gledhill D. (2013). Ocean acidification and coral reefs: Effects on breakdown, dissolution, and net ecosystem calcification. Ann. Rev. Mar. Sci..

[3] Hughes T.P., Baird A.H., Bellwood D.R., Card M., Connolly S.R., Folke C., Grosberg R., Hoegh-Guldberg O., Jackson J.B., Kleypas J. (2003). Climate change, human impacts, and the resilience of coral reefs. Science.

[4] Sully S., Burkepile D.E., Donovan M.K., Hodgson G., van Woesik R. (2019). A global analysis of coral bleaching over the past two decades. Nat. Commun..

[5] Unsworth R.K.F., Collier C.J., Henderson G.M., McKenzie L.J. (2012). Tropical seagrass meadows modify seawater carbon chemistry: Implications for coral reefs impacted by ocean acidification. Environ. Res. Lett..

[6] Macreadie P.I., Anton A., Raven J.A., Beaumont N., Connolly R.M., Friess D.A., Kelleway J.J., Kennedy H., Kuwae T., Lavery P.S. (2019). The future of Blue Carbon science. Nat. Commun..

[7] Macreadie P.I., Atwood T.B., Seymour J.R., Fontes M.L.S., Sanderman J., Nielsen D.A., Connolly R.M. (2019). Vulnerability of seagrass blue carbon to microbial attack following exposure to warming and oxygen. Sci. Total Environ..

[8] Lamb J.B., van de Water J.A., Bourne D.G., Altier C., Hein M.Y., Fiorenza E.A., Abu N., Jompa J., Harvell C.D. (2017). Seagrass ecosystems reduce exposure to bacterial pathogens of humans, fishes, and invertebrates. Science.

[9] Fraser M.W., Gleeson D.B., Grierson P.F., Laverock B., Kendrick G.A. (2018). Metagenomic Evidence of Microbial Community Responsiveness to Phosphorus and Salinity Gradients in Seagrass Sediments. Front. Microbiol..

[10] Hurtado-McCormick V., Kahlke T., Petrou K., Jeffries T., Ralph P.J., Seymour J.R. (2019). Regional and Microenvironmental Scale Characterization of the *Zostera muelleri* Seagrass Microbiome. Front. Microbiol..

[11] Conte C., Rotini A., Manfra L., D’Andrea M.M., Winters G., Migliore L. (2021). The Seagrass Holobiont: What We Know and What We Still Need to Disclose for Its Possible Use as an Ecological Indicator. Water.

[12] Garcias-Bonet N., Eguiluz V.M., Diaz-Rua R., Duarte C.M. (2021). Host-association as major driver of microbiome structure and composition in Red Sea seagrass ecosystems. Environ. Microbiol..

[13] Ugarelli K., Chakrabarti S., Laas P., Stingl U. (2017). The Seagrass Holobiont and Its Microbiome. Microorganisms.

[14] Cúcio C., Overmars L., Engelen A.H., Muyzer G. (2018). Metagenomic Analysis Shows the Presence of Bacteria Related to Free-Living Forms of Sulfur-Oxidizing Chemolithoautotrophic Symbionts in the Rhizosphere of the Seagrass Zostera marina. Front. Mar. Sci..

[15] Mendes R., Garbeva P., Raaijmakers J.M. (2013). The rhizosphere microbiome: Significance of plant beneficial, plant pathogenic, and human pathogenic microorganisms. FEMS Microbiol. Rev..

[16] Cúcio C., Engelen A.H., Costa R., Muyzer G. (2016). Rhizosphere Microbiomes of European Seagrasses Are Selected by the Plant, But Are Not Species Specific. Front. Microbiol..

[17] Borum J., Pedersen O., Greve T.M., Frankovich T.A., Zieman J.C., Fourqurean J.W., Madden C.J. (2005). The potential role of plant oxygen and sulphide dynamics in die-off events of the tropical seagrass, *Thalassia testudinum*. J. Ecol..

[18] Pedersen M.F., Borum J. (1992). Nitrogen Dynamics of Eelgrass Zostera-Marina during a Late Summer Period of High Growth and Low Nutrient Availability. Mar. Ecol. Prog. Ser..

[19] Agawin N.S.R., Ferriol P., Sintes E., Moya G. (2017). Temporal and spatial variability of in situ nitrogen fixation activities associated with the Mediterranean seagrass *Posidonia oceanica* meadows. Limnol. Oceanogr..

[20] Pereg L.L., Lipkin Y., Sar N. (1994). Different Niches of the *Halophila-Stipulacea* Seagrass Bed Harbor Distinct Populations of Nitrogen-Fixing Bacteria. Mar. Biol..

[21] Brodersen K.E., Siboni N., Nielsen D.A., Pernice M., Ralph P.J., Seymour J., Kuhl M. (2018). Seagrass rhizosphere microenvironment alters plant-associated microbial community composition. Environ. Microbiol..

[22] Shade A., Handelsman J. (2012). Beyond the Venn diagram: The hunt for a core microbiome. Environ. Microbiol..

[23] Ling J., Lin X., Zhang Y., Zhou W., Yang Q., Lin L., Zeng S., Zhang Y., Wang C., Ahmad M. (2018). Community Composition and Transcriptional Activity of Ammonia-Oxidizing Prokaryotes of Seagrass *Thalassia hemprichii* in Coral Reef Ecosystems. Front. Microbiol..

[24] Cao D., Cao W., Yu K., Wu G., Yang J., Su X., Wang F. (2017). Evaluation of anthropogenic influences on the Luhuitou fringing reef via spatial and temporal analyses (from isotopic values). J. Geophys. Res. Ocean..

[25] Yue W.Z., Sun C.C., Shi P., Engel A., Wang Y.S., He W.H. (2018). Effect of temperature on the accumulation of marine biogenic gels in the surface microlayer near the outlet of nuclear power plants and adjacent areas in the Daya Bay, China. PLoS ONE.

[26] Zhu Y., Yang S.Z., Ge B.Z., Li Y.X. (2021). Design optimization and uncertainty analysis of multi-energy complementary system for residential building in isolated area. Energy Convers. Manag..

[27] Lu X., Liu Y.W., Zhao H.T., Wang Z.Y. (2020). Corrosion Behavior of Brass H62 in Harsh Marine Atmosphere in Nansha Islands, China. J. Mater. Eng. Perform..

[28] Lakshmanan V., Ray P., Craven K.D. (2017). Rhizosphere Sampling Protocols for Microbiome (16S/18S/ITS rRNA) Library Preparation and Enrichment for the Isolation of Drought Tolerance-Promoting Microbes. Methods Mol. Biol..

[29] Edwards J., Johnson C., Santos-Medellin C., Lurie E., Podishetty N.K., Bhatnagar S., Eisen J.A., Sundaresan V. (2015). Structure, variation, and assembly of the root-associated microbiomes of rice. Proc. Natl. Acad. Sci. USA.

[30] Sun Y., Song Z., Zhang H., Liu P., Hu X. (2020). Seagrass vegetation affect the vertical organization of microbial communities in sediment. Mar. Environ. Res..

[31] Li Y., Hu X., Yang S., Zhou J., Zhang T., Qi L., Sun X., Fan M., Xu S., Cha M. (2017). Comparative Analysis of the Gut Microbiota Composition between Captive and Wild Forest Musk Deer. Front. Microbiol..

[32] Liu Y.X., Qin Y., Chen T., Lu M., Qian X., Guo X., Bai Y. (2020). A practical guide to amplicon and metagenomic analysis of microbiome data. Protein Cell.

[33] Quast C., Pruesse E., Yilmaz P., Gerken J., Schweer T., Yarza P., Peplies J., Glockner F.O. (2013). The SILVA ribosomal RNA gene database project: Improved data processing and web-based tools. Nucleic Acids Res..

[34] Katoh K., Standley D.M. (2013). MAFFT multiple sequence alignment software version 7: Improvements in performance and usability. Mol. Biol. Evol..

[35] Castresana J. (2000). Selection of conserved blocks from multiple alignments for their use in phylogenetic analysis. Mol. Biol. Evol..

[36] Price M.N., Dehal P.S., Arkin A.P. (2009). FastTree: Computing large minimum evolution trees with profiles instead of a distance matrix. Mol. Biol. Evol..

[37] Chen H., Boutros P.C. (2011). VennDiagram: A package for the generation of highly-customizable Venn and Euler diagrams in R. BMC Bioinform..

[38] Anderson M.J. (2001). A new method for non-parametric multivariate analysis of variance. Austral Ecol..

[39] Clarke K.R. (1993). Non-parametric multivariate analyses of changes in community structure. Austral Ecol..

[40] Louca S., Parfrey L.W., Doebeli M. (2016). Decoupling function and taxonomy in the global ocean microbiome. Science.

[41] Deng Y., Jiang Y.H., Yang Y.F., He Z.L., Luo F., Zhou J.Z. (2012). Molecular ecological network analyses. BMC Bioinform..

[42] Luo F., Zhong J.X., Yang Y.F., Scheuermann R.H., Zhou J.Z. (2006). Application of random matrix theory to biological networks. Phys. Lett. A.

[43] Jia X., Li X.D., Zhao Y.H., Wang L., Zhang C.Y. (2019). Soil microbial community structure in the rhizosphere of *Robinia pseudoacacia* L. seedlings exposed to elevated air temperature and cadmium-contaminated soils for 4 years. Sci. Total Environ..

[44] Li J., Luo Z., Zhang C., Qu X., Chen M., Song T., Yuan J. (2020). Seasonal Variation in the Rhizosphere and Non-Rhizosphere Microbial Community Structures and Functions of Camellia yuhsienensis Hu. Microorganisms.

[45] Sun F., Zhang X., Zhang Q., Liu F., Zhang J., Gong J. (2015). Seagrass (*Zostera marina*) Colonization Promotes the Accumulation of Diazotrophic Bacteria and Alters the Relative Abundances of Specific Bacterial Lineages Involved in Benthic Carbon and Sulfur Cycling. Appl. Environ. Microbiol..

[46] Spring S., Bunk B., Sproer C., Rohde M., Klenk H.P. (2018). Genome biology of a novel lineage of planctomycetes widespread in anoxic aquatic environments. Environ. Microbiol..

[47] Zhang X.L., Zhang Q.Q., Yang A.J., Hou L.J., Zheng Y.L., Zhai W.D., Gong J. (2018). Incorporation of Microbial Functional Traits in Biogeochemistry Models Provides Better Estimations of Benthic Denitrification and Anammox Rates in Coastal Oceans. J. Geophys. Res. Biogeosci..

[48] Zhang Y., Ling J., Yang Q., Wen C., Yan Q., Sun H., Van Nostrand J.D., Shi Z., Zhou J., Dong J. (2015). The functional gene composition and metabolic potential of coral-associated microbial communities. Sci. Rep..

[49] Jiang Y.F., Ling J., Wang Y.S., Chen B., Zhang Y.Y., Dong J.D. (2015). Cultivation-dependent analysis of the microbial diversity associated with the seagrass meadows in Xincun Bay, South China Sea. Ecotoxicology.

[50] Park S., Yoshizawa S., Kogure K., Yokota A. (2011). *Rubricoccus marinus* gen. nov., sp nov., of the family ‘Rhodothermaceae’, isolated from seawater. Int. J. Syst. Evol. Microbiol..

[51] Ugarelli K., Laas P., Stingl U. (2019). The Microbial Communities of Leaves and Roots Associated with Turtle Grass (*Thalassia testudinum*) and Manatee Grass (*Syringodium filliforme*) are Distinct from Seawater and Sediment Communities, but Are Similar between Species and Sampling Sites. Microorganisms.

[52] Brodersen K.E., Nielsen D.A., Ralph P.J., Kuhl M. (2015). Oxic microshield and local pH enhancement protects *Zostera muelleri* from sediment derived hydrogen sulphide. New Phytol..

[53] Martin B.C., Bougoure J., Ryan M.H., Bennett W.W., Colmer T.D., Joyce N.K., Olsen Y.S., Kendrick G.A. (2018). Oxygen loss from seagrass roots coincides with colonisation of sulphide-oxidising cable bacteria and reduces sulphide stress. ISME J..

[54] Kuypers M.M. (2015). Microbiology: A division of labour combined. Nature.

[55] Radecker N., Pogoreutz C., Voolstra C.R., Wiedenmann J., Wild C. (2015). Nitrogen cycling in corals: The key to understanding holobiont functioning?. Trends Microbiol..

[56] Welsh D.T. (2000). Nitrogen fixation in seagrass meadows: Regulation, plant-bacteria interactions and significance to primary productivity. Ecol. Lett..

[57] van Kessel M.A., Speth D.R., Albertsen M., Nielsen P.H., Op den Camp H.J., Kartal B., Jetten M.S., Lucker S. (2015). Complete nitrification by a single microorganism. Nature.

[58] Daims H., Lebedeva E.V., Pjevac P., Han P., Herbold C., Albertsen M., Jehmlich N., Palatinszky M., Vierheilig J., Bulaev A. (2015). Complete nitrification by Nitrospira bacteria. Nature.

[59] Schlesner H. (2015). Blastopirellula. Bergey’s Manual of Systematics of Archaea and Bacteria.

[60] More K.D., Giosan L., Grice K., Coolen M.J.L. (2019). Holocene paleodepositional changes reflected in the sedimentary microbiome of the Black Sea. Geobiology.

[61] Martin B.C., Alarcon M.S., Gleeson D., Middleton J.A., Fraser M.W., Ryan M.H., Holmer M., Kilminster K. (2019). Root microbiomes as indicators of seagrass health. FEMS Microbiol. Ecol..

[62] Moriarty D.J.W., Pollard P.C. (1982). Diel Variation of Bacterial Productivity in Seagrass (*Zostera-Capricorni*) Beds Measured by Rate of Thymidine Incorporation into DNA. Mar. Biol..

[63] Holmer M., Duarte C.M., Boschker H.T.S., Barron C. (2004). Carbon cycling and bacterial carbon sources in pristine and impacted Mediterranean seagrass sediments. Aquat. Microb. Ecol..

[64] Sogin E., Michellod D., Gruber-Vodicka H., Bourceau P., Geier B., Meier D., Seidel M., Hach P.F., Procaccini G., Dubilier N. (2019). Seagrass excretes sugars to their rhizosphere making them the sweet spots in the sea. bioRxiv.

