# Supplementary material for: Spatial and Species Variations of Bacterial Community Structure and Putative Function in Seagrass Rhizosphere Sediment
Source: Life (Basel). 2021 Aug 20;11(8):852. doi: 10.3390/life11080852 (PMC8401270; doi:10.3390/life11080852)
Supplement: Supplementary file 1 [file life-11-00852-s001.zip › life-1295335-supplementary.pdf]

Article

Spatial and Species Variations of Bacterial Community Structure and Putative Function in Seagrass Rhizosphere Sediment

Juan Ling [1,2,3,4], Weiguo Zhou [1,2,3,4], Qingsong Yang [1,2,3,4], Jianping Yin [1,2], Jian Zhang [1,5], Qiuying Peng [1,5], Xiaofang Huang [1,5], Yuhang Zhang [1,5] and Junde Dong [1,2,3,4,*]

[1] CAS Key Laboratory of Tropical Marine Bio-resources and Ecology, Guangdong Provincial Key Laboratory of Applied Marine Biology, South China Sea Institute of Oceanology, Chinese Academy of Sciences, Guangzhou 510301, China; lingjuan@scsio.ac.cn (J.L.); zhouweiguo@scsio.ac.cn (W.Z.); qsyang@scsio.ac.cn (Q.Y.); yjp@scsio.ac.cn (J.Y.); zhangjian196@mails.ucas.ac.cn (J.Z.); pengqiuying18@mails.ucas.edu.cn (Q.P.); huangxiaofang602@126.com (X.H.); zhangyuhang20@mails.ucas.ac.cn (Y.Z.)

[2] Southern Marine Science and Engineering Guangdong Laboratory (Guangzhou), Guangzhou 511458, China

[3] Key Laboratory of Tropical Marine Biotechnology of Hainan Province, Sanya Institute of Oceanology, SCSIO; Sanya National Marine Ecosystem Research Station, Chinese Academy of Sciences, Sanya 572000, China

[4] Innovation Academy of South China Sea Ecology and Environmental Engineering, Chinese Academy of Sciences, Guangzhou 511458, China

[5] College of Earth and Planetary Sciences, University of Chinese Academy of Sciences, Beijing 100049, China

* Correspondence: dongjd@scsio.ac.cn; Tel.: +86-20-8910-7830

Citation: Ling, J.; Zhou, W.; Yang, Q.; Yin, J.; Zhang, J.; Peng, Q.; Huang, X.; Zhang, Y.; Dong, J. Spatial and Species Variations of Bacterial Community Structure and Putative Function in Seagrass Rhizosphere Sediment. *Life* **2021**, *11*, 852. https://doi.org/10.3390/life11080852

Academic Editor: Fabia U. Battistuzzi

Received: 6 July 2021

Accepted: 11 August 2021

Published: 20 August 2021

Publisher's Note: MDPI stays neutral with regard to jurisdictional claims in published maps and institutional affiliations.

Copyright: © 2021 by the authors. Submitted for possible open access publication under the terms and conditions of the Creative Commons Attribution (CC BY) license (http://creativecommons.org/licenses/by/4.0/).

Table S1. The sequences characteristics of the all obtained seagrass rhizosphere samples

Coral reef ecosystems	Sample ID	Raw reads	Resampling depth	Good's coverage
**Daya Bay**	DYH1	40,990	33,980	99.30%
	DYH2	41,929	33,980	99.32%
	DYH3	39,296	33,980	99.31%
**Sanya Bay**	SYT1	56,671	33,980	99.07%
	SYT2	54,384	33,980	99.12%
	SYT3	42,129	33,980	98.99%
**Xisha Islands**	XSC1	60,954	33,980	98.96%
	XSC2	67,941	33,980	99.06%
	XSC3	66,866	33,980	99.02%
	XST1	52,168	33,980	99.13%
	XST2	59,573	33,980	98.99%
	XST3	61,669	33,980	99.11%
	XSH1	60,647	33,980	99.01%
	XSH2	55,494	33,980	99.02%
	XSH3	63,638	33,980	99.03%
	XSC1	64,324	33,980	99.14%
	XSC2	60,165	33,980	99.06%
	XSC3	69,321	33,980	99.02%
**Nansha Islands**	NSH1	68,758.	33,980	99.05%
	NSH2	69,867	33,980	99.16%
	NSH3	68,918	33,980	99.10%
	NST1	35,823	33,980	99.16%
	NST2	36,958	33,980	99.20%
	NST3	33,987	33,980	99.23%

Table S2. The significance of differences in taxonomy structure of microbial community between different seagrass species and seagrass *T. hemprichii* and *H. ovalis* from different sampling sites on the genus level

Genus	XSH–XSS	XST–XSH	XSC–XSS	XST–XSS	XSH–XSS	XST–XSC
Bacillus	0.0904	0.2509	0.0904	0.0117*	0.749	0.0007***
Unclassified Desulfobulbaceae	0.0018**	0.7373	0.0018**	0.1672	0.0022**	0.484
Unclassified Rhodobacteraceae	0***	0.455	0***	0.6376	0.5732	0***
Unclassified Bacillaceae2	0.1425	0.3012	0.1425	0.0246*	0.7447	0.0037**
Lactococcus	0.0934	0.2651	0.0934	0.0154*	0.589	0.0009***
Unclassified Desulfobacteraceae	0.0003***	0.4841	0.0003***	0.9566	0.2523	0.0069**
Unclassified Gammaproteobacteria	0.7368	0.2962	0.7368	0.002**	0.0119*	0.0021**
Unclassified Bacillaceae1	0.5845	0.3944	0.5845	0.0401*	0.3919	0.0033**
Desulfopila	0.7858	0.9306	0.7858	0.4427	0.4277	0.5615
Unclassified Chloroflexi	0.0166*	0.1042	0.0166*	0.0739	0.3986	0.0049**
Unclassified Actinobacteria	0.1762	0.033*	0.1762	0.0489*	0.0009***	0.2209
Unclassified Flavobacteriaceae	0.0124*	0.9839	0.0124*	0.0047**	0.0091**	0.0161*
Unclassified Bacteroidetes	0.0221*	0.1013	0.0221*	0.1726	0.3916	0.0191*
Unclassified Anaerolineaceae	0.4537	0.3712	0.4537	0.0899	0.7356	0.0291*
Gp23	0.0121*	0.0759	0.0121*	0.0284*	0.4346	0.3217
Actibacter	0.0001***	0.455	0.0001***	0.0363*	0.0134*	0.2008
Unclassified Deltaproteobacteria	0.0006***	0.0826	0.0006***	0.0516	0.0269*	0.0005***
Unclassified Chromatiales	0.0104*	0.7138	0.0104*	0.0011**	0.0166*	0.0004***
Sulfurovum	0***	0.8582	0***	0***	0.1465	0***
Unclassified Myxococcales	0.0913	0.0266*	0.0913	0.4293	0.0617	0.413
Unclassified Ignavibacteriaceae	0.0057**	0.7954	0.0057**	0.331	0.5594	0.0589
Unclassified Spirochaetaceae	0.0255*	0.3537	0.0255*	0.213	0.001**	0.9391
Gp10	0.0055**	0.9134	0.0055**	0.1297	0.0474*	0.2242
Gp21	0.021*	0.4181	0.021*	0.8677	0.3317	0.0062**
Desulfatiglans	0.0001***	0.0743	0.0001***	0.2739	0.0492*	0.0257*
Caldithrix	0.6846	0.2567	0.6846	0.0518	0.0848	0.0605
Unclassified Thioalkalispiraceae	0.7099	0.445	0.7099	0.0163*	0.0206*	0.0173*
Zooshikella	0.1012	0.2861	0.1012	0.0042**	0.0887	0.0048**
Unclassified Planctomycetaceae	0.7568	0.2933	0.7568	0.033*	0.0074**	0.0639
Gp22	0.0035**	0.0448*	0.0035**	0.3546	0.0175*	0.0319*
Unclassified Proteobacteria	0.0387*	0.2097	0.0387*	0.7704	0.0449*	0.0562
Spirochaeta	0.4012	0.8579	0.4012	0.3762	0.1655	0.2763
Unclassified Cytophagales	0.004**	0.0615	0.004**	0.0152*	0.1813	0.238
Robiginitalea	0.0684	0.6396	0.0684	0.0132*	0.2529	0.0002***
Unclassified Bacteroidales	0.6173	0.2174	0.6173	0.1695	0.9449	0.1407
Aquabacterium	0.7616	0.4464	0.7616	0.0439*	0.0344*	0.0467*
Oceanobacillus	0.1061	0.1572	0.1061	0.0135*	0.6601	0.0036**
Gp9	0.0005***	0.2238	0.0005***	0.0128*	0.0002***	0.0577
Unclassified Helicobacteraceae	1	0.1398	1	0.0485*	0.0837	0.0485*
Unclassified Prolixibacteraceae	0.2753	0.9885	0.2753	0.0277*	0.0499*	0.0394*
Latescibacteria genera incertae sedis	0.0012**	0.1555	0.0012**	0.0004***	0.814	0.1009
Eudoraea	1	0.0809	1	1	0.0809	1
Unclassified Flammeovirgaceae	0.015*	0.0553	0.015*	0.0031**	0.0348*	0.1984
Unclassified Syntrophobacterales	0.2508	0.0033**	0.2508	0.0025**	0.0274*	0.0009***
Desulfobulbus	0.1346	0.0883	0.1346	0.0057**	0.5553	0.0013**
Aminicenantes genera incertae sedis	0.01**	0.9808	0.01**	0***	0***	0.0015**
Unclassified Euryarchaeota	0.3486	0.1155	0.3486	0.2168	0.0776	0.1431

Desulfosarcina	0.0863	0.92	0.0863	**_0.0138*_**	**_0.022*_**	**_0.0298*_**
Haliea	0.1432	0.3809	0.1432	**_0.0114*_**	**_0.0487*_**	**_0.0068**_**
Exiguobacterium	**_0.0262*_**	0.2115	**_0.0262*_**	**_0.0167*_**	0.917	**_0.0009***_**

Note: DYH indicates seagrass *Halophila ovalis* from Daya Bay; SYT indicates seagrass *Thalassia hemprichii* from Sanya Bay, XST, XSH, XSC, and XSS indicate *T. hemprichii*, *H. ovalis*, *Cymodocea nodos* and *Syringodium isoetifolium* collected from the Xisha Islands, and NSH and NST indicates seagrass *H. ovalis* and *T. hemprichii* collected from the Nansha Islands; Values in the table represent p values (* $p < 0.05$, ** $p < 0.01$, and *** $p < 0.001$)

Table S3. The significance of differences in taxonomy structure of microbial community between seagrass *H. ovalis* from different sampling sites on the genus level.

Genus	XS–NS	NS–SY	XS–DY
Bacillus	***0.0217****	***0.0001*****	0.06
Unclassified Desulfobulbaceae	***0.0072*****	***0.0062*****	***0.0113****
Unclassified Rhodobacteraceae	***0.0013*****	0.8764	***0.001******
Unclassified Bacillaceae2	***0.0053*****	0.0302*	0.2196
Lactococcus	***0.0241****	***0.01****	***0.0238****
Unclassified Desulfobacteraceae	0.2376	***0.0103****	***0.0095*****
Unclassified Gammaproteobacteria	***0******	***0.0001******	***0.0021*****
Unclassified Bacillaceae1	***0.0076*****	***0.0298****	0.1974
Desulfopila	0.0614	0.1807	0.9682
Unclassified Chloroflexi	***0.0401****	***0.0255****	***0.0222****
Unclassified Actinobacteria	***0.0162****	***0.0121****	***0.0278****
Unclassified Flavobacteriaceae	0.0007***	0.4402	0.0174*
Unclassified Bacteroidetes	0.0648	***0.0011*****	***0.001******
Unclassified Anaerolineaceae	***0.0008******	***0.0279****	***0.0021*****
Gp23	***0.047****	***0.0014*****	***0.0086*****
Actibacter	***0.0334****	***0.0002******	***0.0019*****
Unclassified Deltaproteobacteria	0.0541	***0.0469****	***0.0112****
Unclassified Chromatiales	***0.0071*****	***0.0043*****	0.2486
Sulfurovum	***0.0322****	***0.0017*****	0.0662
Unclassified Myxococcales	***0.0001******	0.0886	***0.0216****
Unclassified Ignavibacteriaceae	***0.0105****	***0.0011*****	0.0694
Unclassified Spirochaetaceae	***0.0034*****	***0.0047*****	0.0973
Gp10	***0.0021*****	0.1651	***0.0098*****
Gp21	0.1402	***0.0022*****	***0.0069*****
Desulfatiglans	***0.0267****	***0.0143****	***0.0107****
Caldithrix	***0.0224****	***0.0111****	***0.0003******
Unclassified Thioalkalispiraceae	***0.0029*****	***0.0007******	0.0742
Zooshikella	***0.0177****	0.2157	***0.0047*****
Unclassified Planctomycetaceae	***0.0029*****	0.0944	0.2385
Gp22	***0.0061*****	***0.0002******	***0.0017*****
Unclassified Proteobacteria	***0.0279****	0.1467	0.69430
Spirochaeta	***0.0001******	***0.0002******	***0.0096*****
Unclassified Cytophagales	0.2869	***0.0043*****	***0.0008******
Robiginitalea	0.0898	***0.0019*****	***0.0132****
Unclassified Bacteroidales	***0.0035*****	***0******	***0.0014*****
Aquabacterium	***0.0016*****	***0.0015*****	***0.0015*****
Oceanobacillus	0.1159	0.0935	0.3783
Gp9	***0.0005******	***0.0003******	0.1839
Unclassified Helicobacteraceae	***0.0279****	***0.03****	0.0955
Unclassified Prolixibacteraceae	***0.0071*****	***0.008*****	0.2985
Latescibacteria genera incertae sedis	0.7897	0.0501	***0.0014*****
Eudoraea	NA	NA	NA
Unclassified Flammeovirgaceae	***0.0169****	0.4373	0.1487
Unclassified Syntrophobacterales	0.0847	0.087	0.8124
Desulfobulbus	0.5495	0.3128	0.2921
Aminicenantes genera incertae sedis	***0.0211****	0.4574	***0.0179****
UnclassifiedEuryarchaeota	***0.0069*****	***0.0128****	***0.1296***
Desulfosarcina	***0.0028*****	0.3646	***0.0004******
Haliea	***0.0163****	0.2285	***0******
Exiguobacterium	***0.0028*****	***0.0011*****	0.0636

Note: ND means not available; (DYH indicates seagrass *Halophila ovalis* from Daya Bay; SYT indicates seagrass *Thalassia hemprichii* from Sanya Bay, XST, XSH, XSC, and XSS indicate *T. hemprichii*, *H. ovalis*, *Cymodocea nodos* and *Syringodium isoetifolium* collected from the Xisha Islands, and NSH and NST indicates seagrass *H. ovalis* and *T. hemprichii* collected from the Nansha Islands; Values in the table represent p values (* $p < 0.05$, ** $p < 0.01$, and *** $p < 0.001$)

Table S4. The significance of differences in taxonomy structure of microbial community between seagrass *T. hemprichii* from different sampling sites on the genus level.

Genus	XS–DY	NS–DY	XS–NS
Bacillus	***0.0012****	***0.0011****	***0.0083****
Unclassified Desulfobulbaceae	0.0961	***0.0148****	0.1669
Unclassified Rhodobacteraceae	***0.0004*****	***0.0037****	***0.0461****
Unclassified Bacillaceae 2	***0.0012****	***0.0019****	***0.0006*****
Lactococcus	***0*****	***0.0008*****	***0.0013****
Unclassified Desulfobacteraceae	***0.0037****	***0.019****	***0.0055****
Unclassified Gammaproteobacteria	***0*****	0.0758	***0.0001*****
Unclassified Bacillaceae1	***0*****	*******	***0.0003*****
Desulfopila	***0.0153****	***0.0021****	***0.0024****
Unclassified Chloroflexi	0.1893	***0.002****	***0.0059****
Unclassified Actinobacteria	***0.0009*****	***0.1144***	***0.0033****
Unclassified Flavobacteriaceae	***0.0044****	*******	***0*****
Unclassified Bacteroidetes	***0.0001*****	***0.0017****	***0.0065****
Unclassified Anaerolineaceae	***0*****	***0.0001*****	***0.0002*****
Gp23	***0.0004*****	***0.0041****	***0.0015****
Actibacter	***0.0142****	***0.0062****	***0.0028****
Unclassified Deltaproteobacteria	***0.0009*****	0.0602	***0.0005*****
Unclassified Chromatiales	***0*****	0.1776	***0.0009*****
Sulfurovum	***0.0015****	***0.0014****	***0.0007*****
Unclassified Myxococcales	***0.0002*****	0.0812	***0.001*****
Unclassified Ignavibacteriaceae	***0.0309****	***0.0245****	0.0627
Unclassified Spirochaetaceae	***0.005****	***0.0403****	***0.0279****
Gp10	***0.0004*****	0.1867	***0*****
Gp21	***0.0032****	***0.0093****	***0.001****
Desulfatiglans	***0.0045****	***0.0033****	***0.0165****
Caldithrix	***0.0006*****	***0.0007*****	***0.0164****
Unclassified Thioalkalispiraceae	***0*****	***0.003****	***0.0014****
Zooshikella	***0.0121****	***0.0122****	0.4226
Unclassified Planctomycetaceae	***0.0041****	***0.0001*****	0.335
Gp22	***0.0053****	0.2157	***0.0004*****
Unclassified Proteobacteria	***0.0015****	0.0552	0.0981
Spirochaeta	***0.0027****	***0.007****	0.1369
Unclassified Cytophagales	***0.0063****	***0.0253****	***0.0107****
Robiginitalea	***0.0193****	***0.0106****	***0.0008*****
Unclassified Bacteroidales	***0.0003*****	***0.0001*****	0.1061
Aquabacterium	***0.0124****	***0.011****	***0.0055****
Oceanobacillus	***0.0251****	***0.0066****	0.0507
Gp9	***0.0012****	***0.0116****	***0.0474****
Unclassified Helicobacteraceae	***0.0083****	***0.0083****	1
Unclassified Prolixibacteraceae	***0.0077****	***0.0113****	***0.0057****
Latescibacteria genera incertae sedis	***0.0001*****	***0.0002*****	0.1574
Eudoraea	***0.001*****	***0.001*****	1
Unclassified Flammeovirgaceae	***0.0032****	0.0664	***0.011****
Unclassified Syntrophobacterales	***0.0001*****	0.261	***0.0177****
Desulfobulbus	***0.0267****	***0.0246****	0.1649
Aminicenantes genera incertae sedis	0.4785	***0.0017****	***0.0029****
Unclassified Euryarchaeota	***0.0052****	***0.0058****	***0.0003*****
Desulfosarcina	0.0675	0.079	0.0509
Haliea	***0.009****	***0.0117****	***0.041****
Exiguobacterium	***0.0145****	***0.0007*****	***0.0399****

Note: DYH indicates seagrass *Halophila ovalis* from Daya Bay; SYT indicates seagrass *Thalassia hemprichii* from Sanya Bay, XST, XSH, XSC, and XSS indicate *T. hemprichii*, *H. ovalis*, *Cymodocea nodos* and *Syringodium isoetifolium* collected from the Xisha Islands, and NSH and NST indicates seagrass *H. ovalis* and *T.*

hemprichii collected from the Nansha Islands; Values values in the table represent p values (* $p < 0.05$, ** $p < 0.01$, and *** $p < 0.001$).

Table S5. The significance of differences in functional groups of microbial community between different seagrass species on the genus level.

Functional groups	XST–XSC	XSH–XSC	XSS–XSC	XST–XSS	XSH–XSS	XSH–XST
Methanotrophy	0.0628	1	1	0.0628	1	0.0628
Methanogenesis by reduction of methyl compounds with H_2	1	1	0.6856	0.6856	0.6856	1
Hydrogenotrophic methanogenesis	1	1	0.6856	0.6856	0.6856	1
Methanogenesis	1	1	0.6856	0.6856	0.6856	1
Methanol oxidation	0.6495	***0.0322****	***0.0106****	***0.0193****	0.2524	***0.0494****
methylotrophy	0.0533	0.0766	***0.021****	0.0921	0.2619	0.0706
Aerobic ammonia oxidation	0.1235	***0.0248****	***0.0189****	0.2878	***0.0048*****	0.0733
Aerobic nitrite oxidation	0.0728	1	0.4226	0.0765	0.4226	0.0728
Nitrification	0.1164	***0.0248****	***0.0178****	0.264	***0.0048*****	0.071
Sulfate respiration	***0.0161****	0.8032	***0.0025*****	0.0645	***0.0005******	***0.0111****
Sulfur respiration	***0.0055*****	***0.0248****	0.3009	***0.0286****	0.7024	***0.0087*****
Sulfite respiration	0.0514	0.3166	0.1346	0.0661	0.0641	***0.0398****
Respiration of sulfur compounds	***0.0161****	0.8189	***0.0026*****	0.064	***0.0006******	***0.0111****
Chitinolysis	0.0576	0.2567	0.1841	0.4204	1	0.5176
Cellulolysis	***0.0453****	0.2185	0.089	0.0779	0.2619	0.0413*
Xylanolysis	0.0576	0.2567	0.1841	0.4204	1	0.5176
Dark oxidation of sulfur compounds	0.2846	0.1641	1	0.2846	0.1641	0.3235
Fermentation	0.1872	***0.0032*****	0.083	0.0982	***0.002*****	0.9741
Aerobic chemoheterotrophy	***0.0009******	0.6795	***0.0046*****	***0****	***0.0221****	***0.0054*****
Human pathogens all	***0.0168****	***0.0361****	***0.0325****	0.3973	0.1835	0.0559
Animal parasites or symbionts	***0.0168****	***0.0361****	***0.0325****	0.3973	0.1835	0.0559
Aromatic compound degradation	0.3643	0.0587	0.3944	0.1687	0.4957	0.0572
Hydrocarbon degradation	0.0572	0.1835	0.1835	0.0602	1	0.0602
Iron respiration	0.1394	0.0572	0.6495	0.1021	0.2254	0.0634
Nitrate respiration	0.0836	0.1289	***0.0201****	0.3089	0.0594	***0.024****
Nitrate reduction	0.1349	0.1179	***0.0289****	0.5014	0.0552	***0.0278****
Nitrogen respiration	0.0836	0.1289	***0.0201****	0.3089	0.0594	***0.024****
Fumarate respiration	0.1394	0.0572	0.6495	0.1021	0.2254	0.0634
Intracellular parasites	***0.0098*****	0.2124	0.0997	***0.0287****	0.1454	***0.0039*****
Predatory or exoparasitic	***0.0354****	***0.0311****	***0.0133****	***0.0124****	***0.0126****	0.1042
Chloroplasts	0.1728	0.1299	0.2837	***0.026****	***0.0086*****	0.0088**
Anoxygenic photoautotrophy S oxidizing	***0.0388****	***0.0022*****	***0.0083*****	0.1605	***0.0105****	***0.0043*****
Anoxygenic photoautotrophy	***0.0388****	***0.0022*****	***0.0083*****	0.1605	***0.0105****	***0.0043*****
Photoautotrophy	***0.0388****	***0.0022*****	***0.0083*****	0.1605	***0.0105****	***0.0043*****
Phototrophy	***0.0388****	***0.0022*****	***0.0083*****	0.1605	***0.0105****	***0.0043*****
Chemoheterotrophy	***0.0179****	***0.0253****	***0.0016*****	0.3086	***0.0008******	***0.0071*****

Note: DYH indicates seagrass *Halophila ovalis* from Daya Bay; SYT indicates seagrass *Thalassia hemprichii* from Sanya Bay, XST, XSH, XSC, and XSS indicate *T. hemprichii*, *H. ovalis*, *Cymodocea nodos* and *Syringodium isoetifolium* collected from the Xisha Islands, and NSH and NST indicates seagrass *H. ovalis* and *T. hemprichii* collected from the Nansha Islands; Values in the table represent *p* values (* $p < 0.05$, ** $p < 0.01$, and *** $p < 0.001$

Table S6. The significance of differences in functional groups of microbial community between seagrass *T.hemprichii* from different sampling sites on the genus level

Functional groups	XS–DY	NS–DY	XS–NS
Methanotrophy	***0.0064****	***0.0064****	1
Methanogenesis by reduction of methyl compounds with H_2	***0.0064****	***0.0064****	1
Hydrogenotrophic methanogenesis	***0.0064****	***0.0064****	1
Methanogenesis	0.2557	***0.0056****	**0.0023****
Methanol oxidation	***0.0047****	***0.0108****	**0.0061****
methylotrophy	0.1791	0.5788	0.0511
Aerobic ammonia oxidation	1	0.4226	0.4226
Aerobic nitrite oxidation	0.1791	0.5437	0.0621
Nitrification	***0.0002******	***0.0002******	***0.0183****
Sulfate respiration	***0.0048****	0.077	0.2981
Sulfur respiration	***0.0267****	***0.0246****	0.1649
Sulfite respiration	***0.0002******	***0.0002******	0.0182*
Respiration of sulfur compounds	0.3841	0.0824	0.8194
Chitinolysis	***0.0289****	***0.0017****	0.4048
Cellulolysis	0.3841	0.0824	0.8194
Xylanolysis	***0.0069****	0.7127	***0.0231****
Dark oxidation of sulfur compounds	***0******	***0******	***0.0369****
Fermentation	0.2719	***0.0023****	***0.0039****
Aerobic chemoheterotrophy	***0.0057****	***0.0027****	0.0622
Human pathogens all	***0.0057****	***0.0027****	0.0622
Animal parasites or symbionts	1	0.0702	0.0702
Aromatic compound degradation	***0.0005******	***0.0232****	0.1072
Hydrocarbon degradation	0.1835	***0.0445****	***0.0459****
Iron respiration	0.4226	0.077	0.0741
Nitrate respiration	***0.0124****	0.0635	***0.0113****
Nitrate reduction	***0.0086****	***0.0052****	***0.0054****
Nitrogen respiration	***0.0124****	0.0635	***0.0113****
Fumarate respiration	0.4226	0.077	0.0741
Intracellular parasites	0.0515	0.1219	0.5475
Predatory or exoparasitic	0.2031	0.2031	1
Chloroplasts	***0.0105****	***0.0156****	***0.0126****
Anoxygenic photoautotrophy S oxidizing	0.2267	***0.0026****	***0.0027****
Anoxygenic photoautotrophy	0.2267	***0.0026****	***0.0027****
Photoautotrophy	0.2267	***0.0026****	***0.0027****
Phototrophy	0.2267	***0.0026****	***0.0027****
Chemoheterotrophy	***0.0026****	***0.0015****	***0.0467****

Note: DYH indicates seagrass *Halophila ovalis* from Daya Bay; SYT indicates seagrass *Thalassia hemprichii* from Sanya Bay, XST, XSH, XSC, and XSS indicate *T. hemprichii*, *H. ovalis*, *Cymodocea nodos* and *Syringodium isoetifolium* collected from the Xisha Islands, and NSH and NST indicates seagrass *H. ovalis* and *T. hemprichii* collected from the Nansha Islands; Values in the table represent *p* values (* $p < 0.05$, ** $p < 0.01$, and *** $p < 0.001$).

Table S7. The significance of differences in functional groups of microbial community between different seagrass *H.ovalis* from different sampling sites on the genus level.

**Functional group**	**XS vs SY**	**NS vs SY**	**XS vs NS**
Methanotrophy	0.0628	0.4226	0.0642
Methanogenesis by reduction of methyl compounds with H_2	0.4226	***0.0339****	***0.0271****
Hydrogenotrophic methanogenesis	0.4226	***0.0339****	***0.0271****
Methanogenesis	0.4226	***0.0339****	***0.0271****
Methanol oxidation	***0.0201****	***0.0248****	***0.0142****
methylotrophy	0.1574	***0.0024*****	0.2212
Aerobic ammonia oxidation	***0.005*****	***0.0059*****	0.3134
Aerobic nitrite oxidation	0.5185	0.7415	0.3486
Nitrification	***0.0055*****	***0.0046*****	0.3047
Sulfate respiration	***0.0116****	***0.0445****	***0.0155****
Sulfur respiration	***0.0011*****	***0.0474****	***0.0034*****
Sulfite respiration	0.2921	0.3128	0.5495
Respiration of sulfur compounds	***0.0116****	***0.0446****	***0.0154****
Chitinolysis	0.0505	0.353	0.1159
Cellulolysis	0.1982	***0.0259****	***0.0255****
Xylanolysis	0.0505	0.353	0.1159
Dark oxidation of sulfur compounds	0.0782	***0.0009******	***0.0008******
Fermentation	0.1204	0.1397	0.0646
Aerobic chemoheterotrophy	***0.0021*****	0.7545	***0.0107****
Human pathogens all	***0.0198****	0.1333	***0.0095*****
Animal parasites or symbionts	***0.0198****	0.1333	***0.0095*****
Aromatic compound degradation	1	0.4226	0.4226
Hydrocarbon degradation	0.0602	***0.029****	0.5421
Iron respiration	0.0584	0.3897	0.0587
Nitrate respiration	0.0634	***0.0198****	0.3785
Nitrate reduction	***0.0268****	0.2846	***0.0277****
Nitrogen respiration	***0.0327****	***0.0104****	***0.0095*****
Fumarate respiration	***0.0268****	0.2846	***0.0277****
Intracellular parasites	0.0634	***0.0198****	0.3785
Predatory or exoparasitic	***0.005*****	***0.0213****	***0.0073*****
Chloroplasts	0.8615	0.0867	0.0766
Anoxygenic photoautotrophy S oxidizing	***0.0233****	0.057	***0.0229****
Anoxygenic photoautotrophy	***0.0312****	***0.004*****	***0.005*****
Photoautotrophy	***0.0312****	***0.004*****	***0.005*****
Phototrophy	***0.0312****	***0.004*****	***0.005*****
Chemoheterotrophy	***0.0312****	***0.004*****	***0.005*****

Note: DYH indicates seagrass *Halophila ovalis* from Daya Bay; SYT indicates seagrass *Thalassia hemprichii* from Sanya Bay, XST, XSH, XSC, and XSS indicate *T. hemprichii*, *H. ovalis*, *Cymodocea nodos* and *Syringodium isoetifolium* collected from the Xisha Islands, and NSH and NST indicates seagrass *H. ovalis* and *T. hemprichii* collected from the Nansha Islands; Values in the table represent p values (* $p < 0.05$, ** $p < 0.01$, and *** $p < 0.001$).

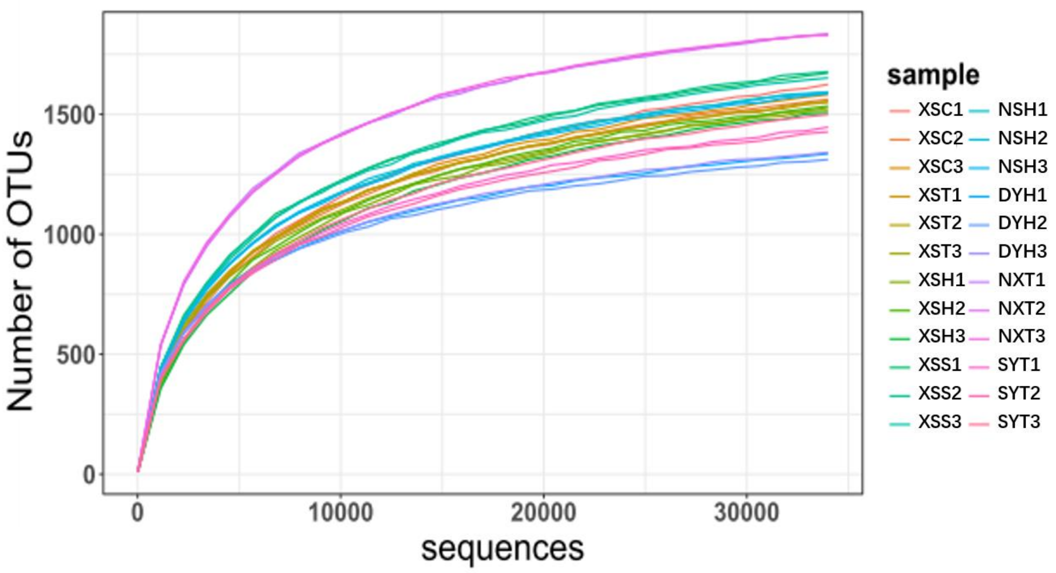

Figure S1 Rarefaction curves of OTU diversity in each sample (n=24) (DYH indicates seagrass *Halophila ovalis* from Daya Bay; SYT indicates seagrass *Thalassia hemprichii* from Sanya Bay, XST, XSH, XSC, and XSS indicate *T. hemprichii*, *H. ovalis*, *Cymodocea nodos* and *Syringodium isoetifolium* collected from the Xisha Islands, and NSH and NST indicates seagrass *H. ovalis* and *T. hemprichii* collected from the Nansha Islands)

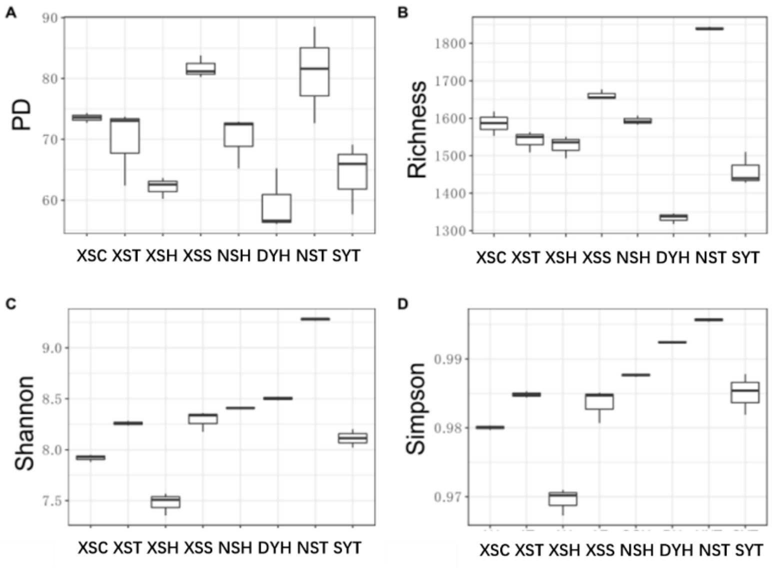

Figure S2. Phylogenetic and taxonomic alpha diversity of seagrass rhizosphere microbial communities (A: phylogenetic diversity; B: richness; C: Shannon; D： Simpson) ((DYH indicates seagrass *Halophila ovalis* from Daya Bay; SYT indicates seagrass *Thalassia hemprichii* from Sanya Bay, XST, XSH, XSC, and XSS indicate *T. hemprichii*, *H. ovalis*, *Cymodocea nodos* and *Syringodium isoetifolium* collected from the Xisha Islands, and NSH and NST indicates seagrass *H. ovalis* and *T. hemprichii* collected from the Nansha Islands))

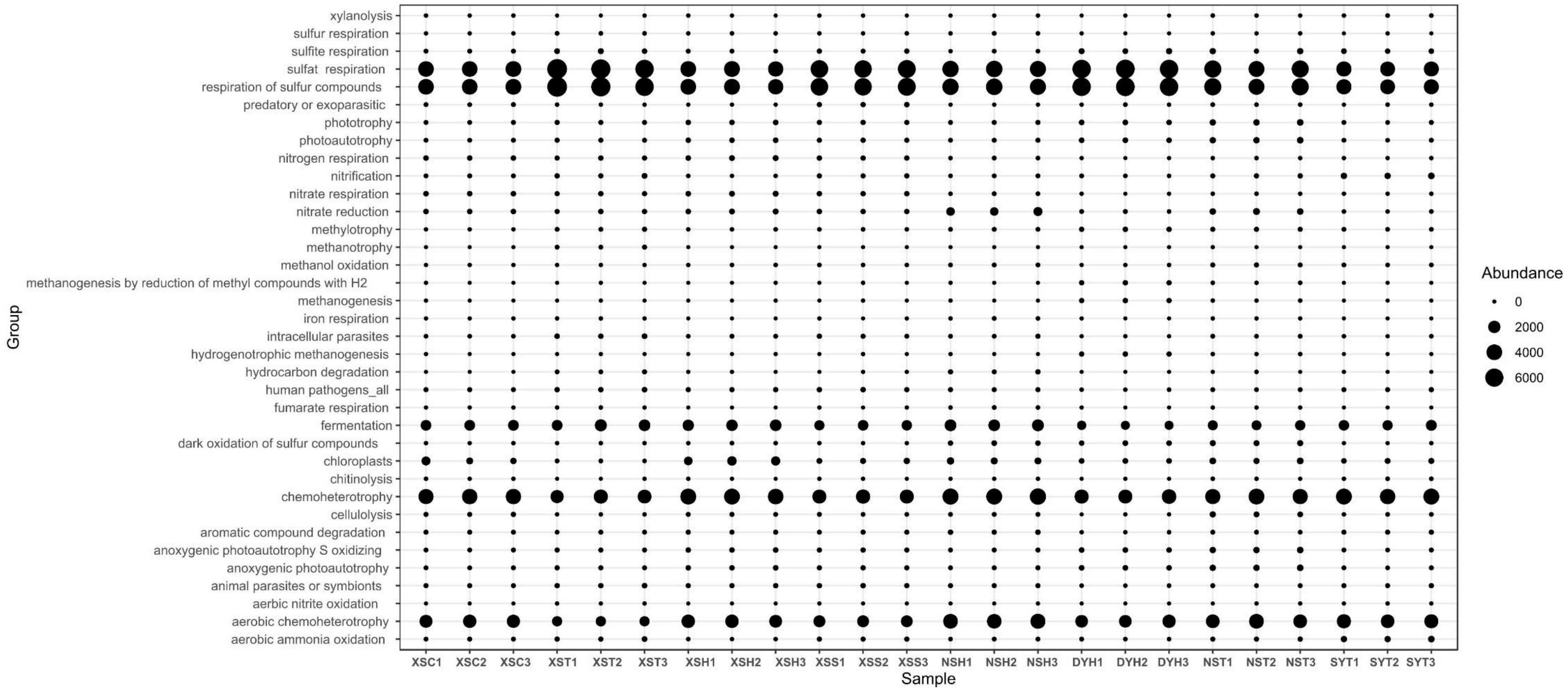

Figure S3. The bubble plot of functional groups in all rhizosphere microbial communities of seagrass (DYH indicates seagrass *Halophila ovalis* from Daya Bay; SYT indicates seagrass *Thalassia hemprichii* from Sanya Bay, XST, XSH, XSC, and XSS indicate *T. hemprichii*, *H. ovalis*, *Cymodocea nodos* and *Syringodium isoetifolium* collected from the Xisha Islands, and NSH and NST indicates seagrass *H. ovalis* and *T. hemprichii* collected from the Nansha Islands)

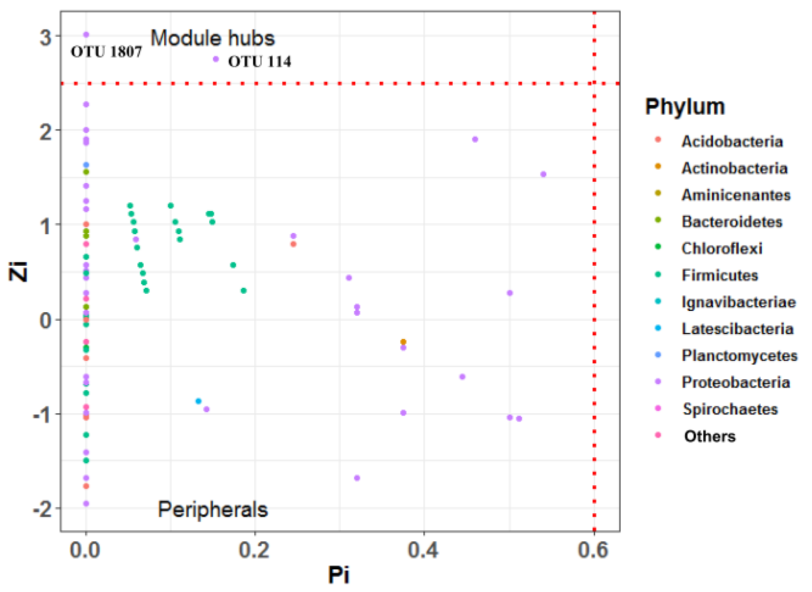

Figure S4. Zi-Pi plot of network nodes of core microbial community shared by all samples ((DYH indicates seagrass *Halophila ovalis* from Daya Bay; SYT indicates seagrass *Thalassia hemprichii* from Sanya Bay, XST, XSH, XSC, and XSS indicate *T. hemprichii*, *H. ovalis*, *Cymodocea nodos* and *Syringodium isoetifolium* collected from the Xisha Islands, and NSH and NST indicates seagrass *H. ovalis* and *T. hemprichii* collected from the Nansha Islands))
